# Interrogation of the microenvironmental landscape in spinal ependymomas reveals dual functions of tumor-associated macrophages

**DOI:** 10.1038/s41467-021-27018-9

**Published:** 2021-11-25

**Authors:** Qianqian Zhang, Sijin Cheng, Yongzhi Wang, Mengdi Wang, Yufeng Lu, Zengqi Wen, Yuxin Ge, Qiang Ma, Youqiao Chen, Yaowu Zhang, Ren Cao, Min Li, Weihao Liu, Bo Wang, Qian Wu, Wenqing Jia, Xiaoqun Wang

**Affiliations:** 1 State Key Laboratory of Brain and Cognitive Science, CAS Center for Excellence in Brain Science and Intelligence Technology, Institute of Brain-Intelligence Technology (Shanghai), Bioland Laboratory (Guangzhou), Institute of Biophysics, Chinese Academy of Sciences, 100101 Beijing, China; 2 University of Chinese Academy of Sciences, 100049 Beijing, China; 3 Department of Neurosurgery, Beijing Tiantan Hospital, Capital Medical University, 100070 Beijing, China; 4 China National Clinical Research Center for Neurological Diseases, 100070 Beijing, China; 5 State Key Laboratory of Cognitive Neuroscience and Learning, IDG/McGovern Institute for Brain Research, Beijing Normal University, 100875 Beijing, China; 6 Chinese Institute for Brain Research, 102206 Beijing, China; 7 Beijing Advanced Innovation Center for Big Data-Based Precision Medicine, Beihang University & Capital Medical University, 100069 Beijing, China; 8 Advanced Innovation Center for Human Brain Protection, Beijing Institute for Brain Disorders, Capital Medical University, 100069 Beijing, China

**Keywords:** CNS cancer, Cancer in the nervous system

## Abstract

Spinal ependymomas are the most common spinal cord tumors in adults, but their intratumoral cellular heterogeneity has been less studied, and how spinal microglia are involved in tumor progression is still unknown. Here, our single-cell RNA-sequencing analyses of three spinal ependymoma subtypes dissect the microenvironmental landscape of spinal ependymomas and reveal tumor-associated macrophage (TAM) subsets with distinct functional phenotypes. *CCL2*^+^ TAMs are related to the immune response and exhibit a high capacity for apoptosis, while *CD44*^+^ TAMs are associated with tumor angiogenesis. By combining these results with those of single-cell ATAC-sequencing data analysis, we reveal that TEAD1 and EGR3 play roles in regulating the functional diversity of TAMs. We further identify diverse characteristics of both malignant cells and TAMs that might underlie the different malignant degrees of each subtype. Finally, assessment of cell-cell interactions reveal that stromal cells act as extracellular factors that mediate TAM diversity. Overall, our results reveal dual functions of TAMs in tumor progression, providing valuable insights for TAM-targeting immunotherapy.

## Abstract (web summary)

The intratumoural heterogeneity of spinal ependymomas and the role of microglia in tumour progression remain underexplored. Here, the authors perform single-cell RNA- and ATAC-sequencing data analysis of three subtypes and reveal tumour-associated macrophage subsets with distinct functional phenotypes.

## Introduction

Ependymal tumors are neuroepithelial malignancies located in both the brain and spinal cord^1^, and spinal ependymomas are the most common spinal cord tumors in adults, comprising ~60% of all intramedullary neoplasms^2^. Surgical resection remains the mainstay of treatment for this disease. Due to the invasive growth of tumor cells, it is difficult to completely remove tumors without obvious tumor margins, and aggressive surgical removal is accompanied by potentially severe sensory and motor dysfunction^3,4^. For patients with subtotal resection who do not receive adjuvant radiotherapy, the recurrence rate of tumors is up to 50–70%^4^. Therefore, there is an urgent need to identify novel therapeutic targets for spinal ependymomas.

In the past decade, studies focused on genomics, transcriptomic, and methylomics have greatly expanded our understanding of the biology underlying human ependymomas. Extensive genomic and transcriptomic analyses uncovered ependymoma oncogenes and tumor suppressor genes^5^ and revealed neural stem cells and glial cells as putative cells of origin^6,7^. The molecular events involving chromosome 22 and mutation of the *NF2* gene have long been known as hallmark genetic aberrations of spinal ependymomas^8^. Genome-wide DNA methylation profiling of ependymomas identified nine molecular subgroups, three in each anatomical compartment of the central nervous system (CNS), including the spinal, posterior fossa, and supratentorial areas, which could help the precise diagnosis and risk stratification of ependymoma patients in the clinic^1^. Within the spinal ependymoma histopathological subtypes, subependymoma (SE) and ependymoma (EPN) are considered as CNS WHO grade I and II with good prognosis, while anaplastic ependymoma (AEP) are considered as grade III showing aggressive identity^9^. The previous studies were informed by bulk tumor samples and largely focused on the developmental origins and candidate driver genes of ependymomas.

Single-cell RNA-seq (scRNA-seq) and single-cell ATAC-seq (scATAC-seq) technologies have been successfully applied to characterize tumor heterogeneity at single-cell resolution^10^ and have revealed the cellular diversity of the tumor microenvironment (TME) in multiple cancer types^11–18^. Recent studies have begun to reveal the cellular heterogeneity of malignant cells in ependymomas by using scRNA-seq^19,20^, with samples mainly collected from childhood brain ependymomas. However, the cellular diversity in spinal ependymomas, especially the complexity of stromal and immune cells, remains less studied. Recent advances in cancer treatments targeting tumor-infiltrating immune cells have emerged^21^. Microglia are the resident macrophages in CNS parenchyma and have emerged as a promising cellular target for tumor immunotherapy considering their roles in neuroinflammation^22^. We speculate that a deep understanding of tumor-associated macrophages would reveal potential therapeutic targets for ependymomas.

In this work, we perform scRNA-seq, scATAC-seq, and bulk epigenetic ChIP-seq analyses of cells isolated from patients diagnosed with spinal cord ependymomas, determining the diversity of malignant cells and immune cells in these spinal cord ependymomas. Two distinct TAM subsets with different ontogenies and dual functional phenotypes could play crucial roles in tumor growth and invasiveness, which may inform TAM-targeting immunotherapy strategies in human ependymomas and other cancers.

## Results

### Intratumoral cell types in spinal ependymomas revealed by scRNA-seq and scATAC-seq

**Fig. 1:**
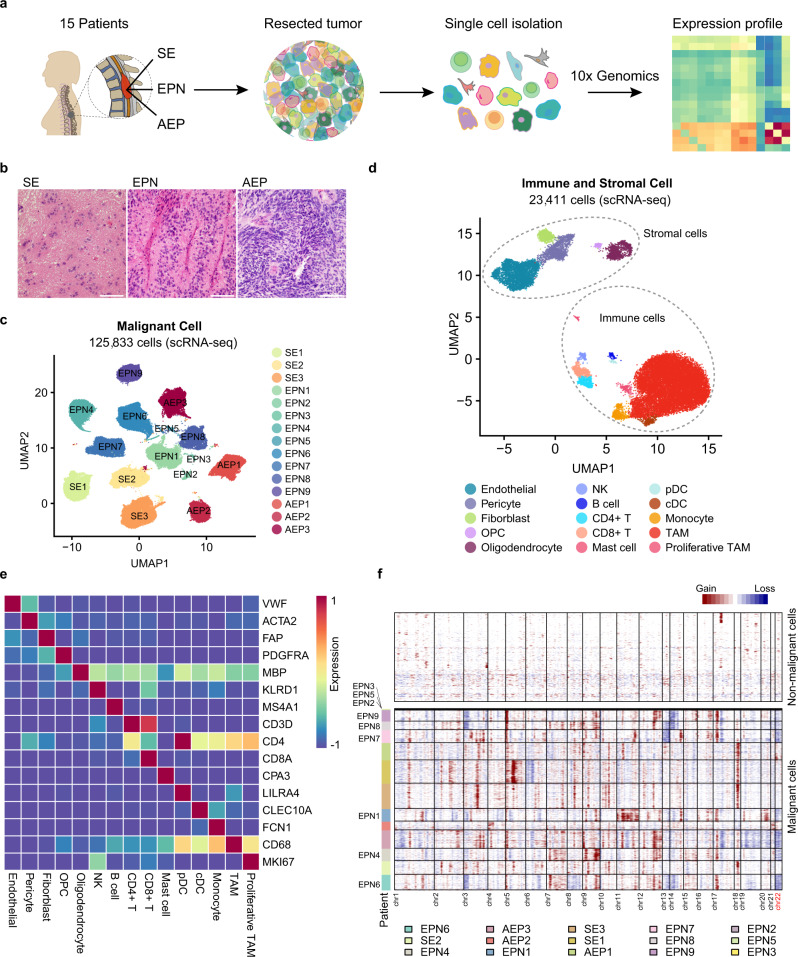
Intratumoral cell types in spinal ependymomas revealed by scRNA-seq. **a** Scheme of the overall study design. scRNA-seq (10× Genomics) was applied to cells isolated from three subtypes of spinal ependymomas. **b** Hematoxylin and eosin (HE) staining of tumors from different cancer subtypes. The scale bar represents 30 μm. Images shown are representatives of more than three samples from three independent experiments. **c** Uniform Manifold Approximation and Projection (UMAP) plot showing the patient distribution of malignant cells, without donor effect correction. **d** UMAP plot of stromal and immune cell types from all patients (SE1-3, AEP1-3, EPN1-9), donor effect corrected by BBKNN. **e** Heatmap showing the expression of canonical gene markers of stromal and immune cell types. **f** Inference of copy-number variations (CNVs) from scRNA-seq. Each row corresponds to a cell. The top panel represents nonmalignant cells, and the bottom panel represents malignant cells, ordered by the patient. See also Supplementary Figs. 1 and 2 and Supplementary Data 1 and 2.

To comprehensively catalog the cellular heterogeneity of spinal ependymomas, we performed droplet-based scRNA-seq (10× Genomics) on cells isolated from tumor tissues of 15 treatment-naive patients diagnosed with different subtypes of spinal ependymoma, including subependymoma (SE, three patients), ependymoma (EPN, 9 patients), and anaplastic ependymoma (AEP, three patients) (Fig. 1a, b and Supplementary Data 1). Notably, SE patients exhibited the lowest degree of malignancy among the three subtypes (Fig. 1b). With strict quality control and filtration (“Methods”), a total of 149,244 cells with a median of 6334 unique molecular identifiers (UMIs) and 2757 genes were retained for downstream analysis (Supplementary Fig. 1a). We first distinguished nonmalignant cells (using canonical gene markers of stromal and immune cells) from malignant cells in the first round of graph-based unsupervised clustering (Supplementary Fig. 1a, b) and found that malignant cells clustered according to tumor origin, while nonmalignant cells clustered by cell type (Fig. 1c and Supplementary Fig. 1c), consistent with previous studies^23–25^. We then separated nonmalignant cells from malignant cells and identified 14 cell types according to the expression of canonical gene markers, including markers of CD8^+^ T cells, CD4^+^ T cells, B cells, mast cells, conventional dendritic cells (cDCs), plasmacytoid dendritic cells (pDCs), monocytes, TAMs, fibroblasts, endothelial cells, and pericytes (Fig. 1d, e, Supplementary Fig. 1d, and Supplementary Data 2). To validate cells classified as malignant, we further inferred large-scale copy-number variations (CNVs) based on scRNA-seq expression profiles with stromal and immune cells as controls^23–27^ and detected large-scale CNVs in malignant cells from each patient but not in nonmalignant cells, including the reported 22q loss^8^ in all nine EPN patients (Fig. 1f), which confirmed the rationality of the unsupervised clustering. We then compared the cell-type percentages in each patient and observed that the abundance of malignant cells and the composition of stromal and immune cells varied across patients (Supplementary Fig. 1e, f), suggesting a considerable degree of tumor heterogeneity. These results provide a basic description of the TME cellular composition of human ependymomas.

We also generated scATAC-seq data from cells isolated from tumor tissues of two AEP patients to capture the chromatin regulatory landscape that governs transcription dynamics in spinal ependymomas with the highest degree of malignancy. In total, 2854 cells with 635,774 reproducible peaks of chromatin accessibility passed strict quality control and were used for the downstream analysis (Supplementary Fig. 2a). We identified five distinct clusters based on unsupervised clustering and annotated their cell types. Two of those clusters exhibited specifically accessible peaks neighboring OPC markers (*OLIG1* and *OLIG2*) and TAM markers (*PTPRC* and *CD68*) (Supplementary Fig. 2b, c). The remaining clusters were regarded as malignant cells that showed high patient specificity and were annotated as astroependymal-like cells, ependymal-like cells, and neuronal-like cells (Supplementary Fig. 2b, c).

### Two TAM subsets show distinct functional phenotypes in ependymomas

**Fig. 2:**
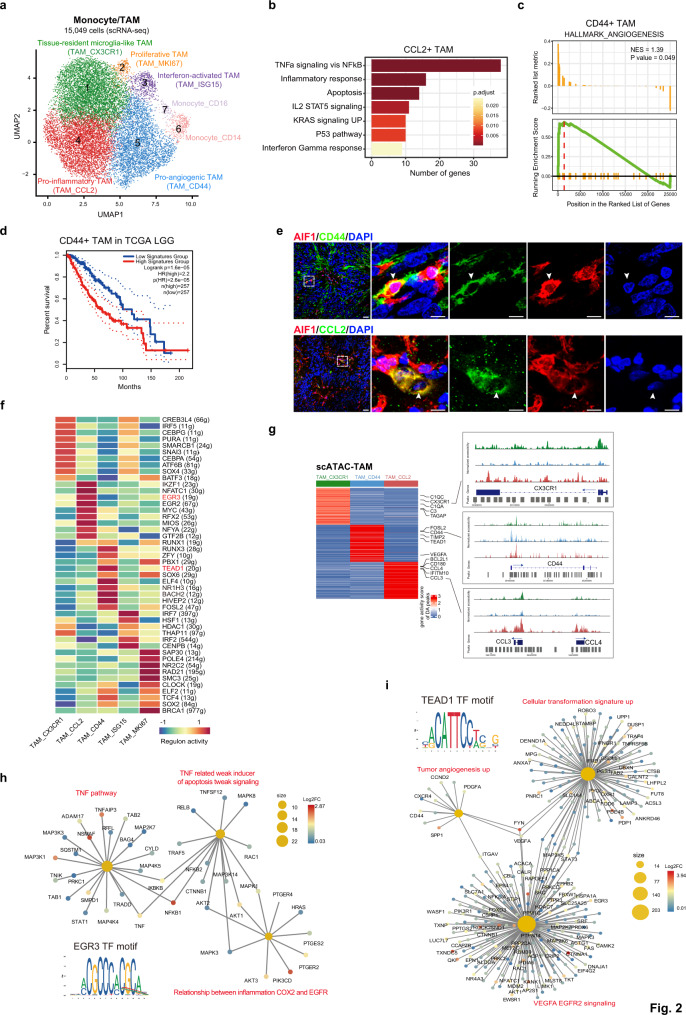
Two TAM subsets show distinct functional phenotypes in ependymomas. **a** UMAP plot of seven subclusters of monocytes and TAMs. **b** Bar plot of enriched hallmark pathways for genes upregulated in *CCL2*^+^ TAMs. *P* values were calculated by using *enrichr* function from R package clusterProfiler with hypergeometric test statistical analyses. Source data are provided as a Source Data file. **c** Enrichment plot of the hallmark angiogenesis pathway in *CD44*^+^ TAMs. *P* values were calculated by using *GESA* function from R package clusterProfiler. **d** Kaplan–Meier plot showing worse clinical outcome for high expression of *CD44*^+^ TAMs signature genes in LGG patients from TCGA. +, censored observations. *P* values were calculated by using both the log-rank test and Cox proportional hazards model. **e** Representative example of an EPN tumor stained by IF. The upper panel image indicates *AIF1*^*+*^*CD44*^*+*^ TAMs (the scale bar represents 30 μm). The dashed boxes highlight regions shown on the right side and the arrow depicts the *CD44*^*+*^ TAMs in fluorescent images (the scale bar represents 100 μm). The bottom panel image indicates *AIF1*^*+*^*CCL2*^*+*^ TAMs (the scale bar represents 30 μm). The dashed boxes highlight regions shown on the right side and the arrow depicts the *CCL2*^*+*^ TAMs in fluorescent images (the scale bar represents 100 μm). Images shown are representatives of three samples from three independent experiments. **f** Heatmap showing TF activity for each TAM subsets. The row name showed the regulon gene sets name and gene number is written in the round brackets. The red color marks the regulon of interest. **g** Heatmap showing cluster-specific ATAC-seq peaks (left). Browser tracks showing ATAC-seq signals for selected marker genes (right). **h** Network plot of enriched curated gene sets for genes regulated by EGR3 in *CCL2*^*+*^ TAM subset. Nodes for genes were colored by log2FC, and the sizes of nodes for enriched pathways were correlated with the number of genes. **i** Network plot of enriched curated gene sets for genes regulated by TEAD1 in *CD44*^*+*^ TAM subset. Nodes for genes were colored by log2FC, and the sizes of nodes for enriched pathways were correlated with the number of genes. See also Supplementary Figs. 2–3 and Supplementary Data 3–5.

We next focused on the heterogeneity of monocytes and TAMs and performed unsupervised graph-based clustering of their transcriptomes, which resulted in seven subclusters with unique signature genes, including two monocyte subsets (classical *CD14*^*+*^ and nonclassical *CD16*^*+*^ monocytes) and five TAM subsets (Fig. 2a, Supplementary Fig. 3a, and Supplementary Data 3). The tissue-resident microglia-like TAMs in cluster 1 (TAM_CX3CR1) were characterized by high expression of the complement gene *C1QC* and the microglia homeostatic gene *CX3CR1*, similar to the reported homeostatic microglia subset in the brain^28^. The proliferative TAMs in cluster 2 (TAM_MKI67) were characterized by cell cycle gene expression, including expression of *MKI67* and *TOP2A*. The interferon-activated TAMs in cluster 3 (TAM_ISG15), which constituted a small population of TAMs, expressed high levels of interferon response genes, such as *ISG15* and *IFIT3*. The pro-inflammatory TAMs in cluster 4 (TAM_CCL2) showed high expression of the pro-inflammatory mediator *IL1B* and CNS inflammation-associated chemokines^29^ (*CCL3, CCL4,* and *CCL2*) (Supplementary Fig. 3b), resembling the pre-activated subset reported in normal brain samples from both humans and mice^28,30,31^. We noticed that the *CCL2*^+^ pro-inflammatory TAMs also exhibited high expression of apoptosis-related genes, such as *PMAIP1* and *NEDD9* (Supplementary Fig. 3b). Enrichment analysis further confirmed that *CCL2*^+^ TAMs showed high activity in both inflammatory response and apoptosis pathways (Fig. 2b and Supplementary Data 4). The remaining TAMs, falling into the pro-angiogenic TAM cluster (TAM_CD44, cluster 5), were characterized by high expression of the angiogenesis-associated genes *CD44*^32^ and *VEGFA*^33^ (Supplementary Fig. 3b). Analysis of the hallmark angiogenesis pathway in *CD44*^+^ TAMs using gene set enrichment analysis (GSEA) revealed a significant enrichment of tumor angiogenesis (Fig. 2c), suggesting a pro-tumorigenic role in ependymomas. We then investigated the relationship of *CD44*^+^ TAMs with patient prognosis and found that higher expression of signature genes of *CD44*^+^ TAMs was associated with a worse clinical outcome in similar cancer types from The Cancer Genome Atlas (TCGA), including brain lower-grade glioma (LGG) and glioblastoma (GBM) (Fig. 2d and Supplementary Fig. 3c). Using immunofluorescence (IF) staining of tumor sections from EPN and AEP patients, we confirmed the presence of *CCL2*^+^ TAMs and *CD44*^+^ TAMs as subgroups (Fig. 2e and Supplementary Fig. 3e) and observed higher proportions of these two TAM subsets in AEP (Supplementary Fig. 3d). Notably, the functional potentiality of each TAM subcluster was implied by their signature genes and additional validation experiments are required to fully elucidate their biological characteristics in the future.

We then examined the regulatory networks that underlie each TAM subset. Using SCENIC^34^, we identified specific transcription factor (TF) regulons in each TAM subset (Fig. 2f). We observed that the IKZF1 and EGR3 regulons, which are related to regulating inflammatory genes^35,36^, were highly activated in *CCL2*^+^ TAMs (Fig. 2f). The activated TEAD1 regulon was specifically found in *CD44*^+^ TAMs (Fig. 2f), and *TEAD1* has been reported to directly promote human glioblastoma cell migration^37^ and regulate pro-angiogenic activity through YAP1-TEAD1-PGC1α signaling in endothelial cells^38^. To complement the regulons predicted from the scRNA-seq data, we further analyzed the scATAC-seq data of TAMs. Using the TAM subpopulations derived from the scRNA-seq data as a reference, we identified three TAM subpopulations (TAM_CX3CR1, TAM_CCL2, TAM_CD44) with the scATAC-seq data, and each subpopulation exhibited specific differentially accessible (DA) sites, which could affect marker genes of TAM subpopulations (Fig. 2g, Supplementary Fig. 2d, e, and Supplementary Data 5). We reasoned that due to the limited number of captured cells, other TAM subclusters were missed in our scATAC-seq data. We then applied motif enrichment analysis to cluster-specific accessible peaks to determine potentially key TFs in each TAM subcluster. Strikingly, we observed that the binding motif of TEAD1 was enriched in *CD44*^+^ TAMs and that one of its binding sites was located close to the transcription start site (TSS) of *VEGFA* (Supplementary Fig. 2f). Furthermore, TEAD1 was found to be the upstream regulator of several signaling pathways, including tumor angiogenesis, the cell transformation signature, and VEGFA-EGFR2 signaling (Fig. 2i), indicating its possible role in driving the pro-angiogenic phenotype. For *CCL2*^+^ TAMs, we uncovered enrichment of the EGR3 motif, whose target genes were related to the regulation of TNF-related weak inducer of apoptosis (TWEAK) signaling and the TNF pathway (Fig. 2h), suggesting the important role of EGR3 in promoting the antitumor phenotype of *CCL2*^+^ TAMs. Overall, our scRNA-seq and scATAC-seq analyses unearthed plausible regulatory mechanisms that shape the functional diversity of TAMs in ependymoma.

**Fig. 3:**
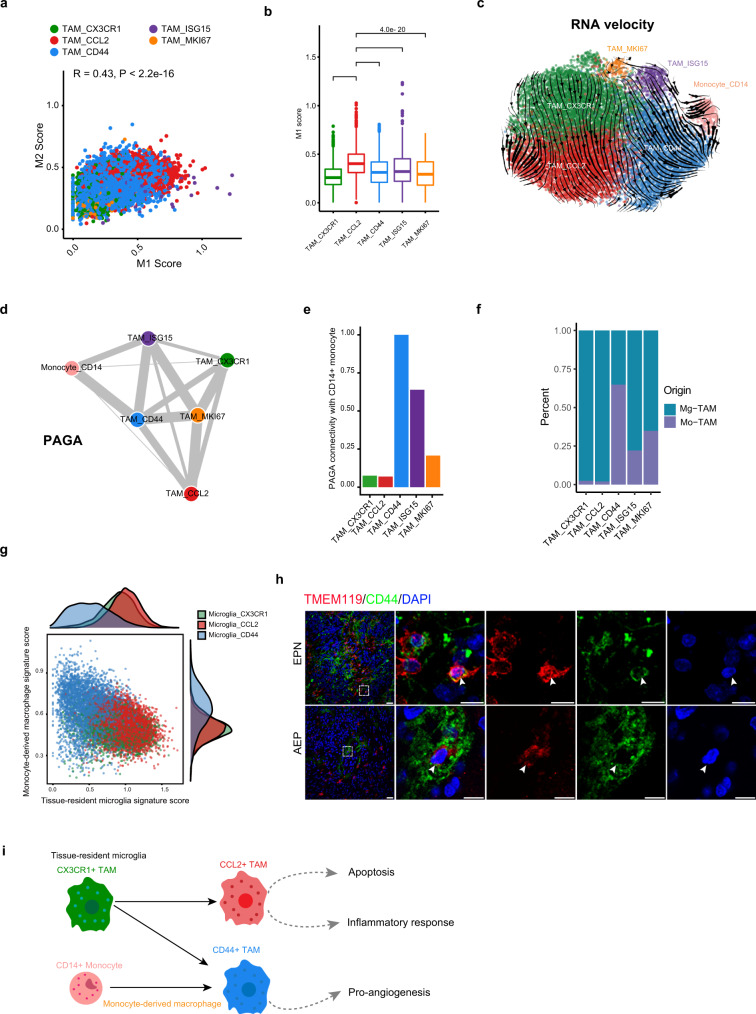
Developmental trajectory of TAM subsets in spinal ependymomas. **a** Scatter plot showing the Pearson correlation between the M1 and M2 signature scores. **b** Box plot showing the M1 signature score in each TAM subset. *P* values were calculated by the Wilcoxon test, two-sided comparisons. Multiple hypothesis correction using the Benjamini–Hochberg procedure. *n* = 15,049 cells. The center line, bounds of box, and whiskers represent mean, 25th to 75th percentile range, and minimum to maximum range in all boxplots. **c** Steady-state RNA velocity of TAM subsets. **d** PAGA graph showing the inferred developmental trajectories for TAM subsets. The edge width was correlated with the strength of connectivity between two subclusters. **e** Bar plot showing PAGA connectivity with CD14^+^ monocytes. **f** Bar plot showing the proportion of monocyte origin and tissue-resident microglia origin across each TAM subset (using data reported by Pombo et al. as a reference). Source data are provided as a Source Data file. **g** Scatter plot showing the scores by average expression of signature genes of tissue-resident microglia versus monocyte-derived macrophages. **h** Representative examples of tumor section stained by IF. The upper panel image indicates *CD44*^*+*^ *TMEM119*^+^ microglia in EPN tumor (the scale bar represents 30 μm). The dashed boxes highlight regions shown on the right side and the arrow depicts the *CD44*^*+*^ microglia in fluorescent images (the scale bar represents 100 μm). The bottom panel image indicates *CD44*^*+*^ *TMEM119*^+^ microglia in AEP tumor (the scale bar represents 30 μm). The dashed boxes highlight regions shown on the right side and the arrow depicts the *CD44*^*+*^ microglia in fluorescent images (the scale bar represents 100 μm). Images shown are representatives of three samples from three independent experiments. **i** Model of the developmental trajectory of monocyte/TAM lineages in spinal ependymomas. See also Supplementary Figs. 3 and 4.

We next examined the expression pattern of the M1 and M2 macrophage signatures in these TAM subsets and observed a coexpression pattern (Fig. 3a). Consistent with previous studies^13,17,18,39^ and a recent pan-cancer analysis of TAMs^40^, our results indicated that such a simple macrophage polarization model defined in vitro was also not suitable for TAMs in CNS tumors. We reasoned that the complicated stimuli that coexist within the TME, rather than the one-way stimulus implied by the in vitro model, induce the complex TAM activation status in tumors^41–43^. Notably, we found that *CCL2*^+^ microglia exhibited the highest M1 signature score, suggesting their stronger antitumor capacity (Fig. 3b).

Studies have suggested that TAMs can originate from both tissue-resident macrophages called microglia and tissue-invading monocytes in brain tumors^44,45^. To explore the ontogenies of TAM subsets in spinal ependymomas, we employed RNA velocity analysis and identified two different origins of *CD44*^+^ TAMs (Fig. 3c), including both *CD14*^*+*^ monocytes and *CX3CR1*^+^ TAMs. In contrast, our velocity analysis revealed that *CCL2*^+^ TAMs originated solely from *CX3CR1*^+^ TAMs (Fig. 3c). To complement this prediction, we performed PAGA analysis^46^. As expected, we observed that *CX3CR1*^+^ TAMs and *CCL2*^+^ TAMs showed much lower connectivity with CD14^+^ monocytes (Fig. 3d, e), implying that they were more likely to be TRMs or their derivatives. Moreover, *CD44*^+^ TAMs were predicted to have strong connectivity with both *CX3CR1*^+^ TAMs and *CD14*^*+*^ monocytes (Fig. 3d). We used SingleR with data from Pombo et al.^47^ as a reference to predict the origin of TAMs in ependymoma. Consistently, *CD44*^*+*^ TAMs were predicted to have two diverse origins, and *CCL2*^+^ TAMs mainly developed from tissue-resident microglia (Fig. 3f). Scoring of cells according to the expression of tissue-resident microglia-specific versus monocyte-derived macrophage-specific genes collected from human brain gliomas^48^ further supported the diverse ontogenies of the two TAM subsets (Fig. 3g). With immunostaining, we confirmed the presence of CD44^+^ microglia (also TMEM119^+^) in EPN and AEP tumor sections, indicating that some *CD44*^*+*^ TAMs were tissue-resident microglia or their derivatives (Fig. 3h). However, fully elucidating the complex trajectories of TAMs in the TME requires additional lineage tracing studies. Collectively, our extensive analyses indicated that *CD44*^+^ TAMs might develop from both *CD14*^*+*^ monocytes and *CX3CR1*^+^ TRMs, while *CCL2*^+^ TAMs might develop from *CX3CR1*^+^ TRMs (Fig. 3i).

For a comprehensive description of TAMs in ependymoma, we compared TAMs in our study with microglia in the normal brain^49^ and TAMs in glioblastoma (GBM)^47,50^ and IDH-mutant high-grade gliomas (HGG)^51^. We noticed that microglia from the normal brain showed high expression of *CCL2* and *CCL3*, resembling tissue-resident *CCL2*^*+*^ TAMs in ependymoma (Supplementary Fig. 3f, g). For TAMs from other neurological tumors, we observed that the *CCL2*^+^ TAM cluster was present in both GBM and HGG, while the *CD44*^+^ TAM cluster was only present in GBM (Supplementary Fig. 4a, b, d). Furthermore, integrated analysis of TAMs from GBM and spinal ependymoma revealed that the *CD44*^+^ TAMs from the two tumors were closely related to each other, but they did not overlap in the UMAP plot, indicating that they had their own characteristics in each tumor (Supplementary Fig. 4c, e). Taken together, our analysis results indicated that *CD44*^*+*^ TAMs were not specific to EPNs and could be present in other but not all neurological tumors, which may be related to the diverse tumor microenvironments of different cancer types.

### Complex expression heterogeneity of malignant cells in each ependymoma subtype

**Fig. 4:**
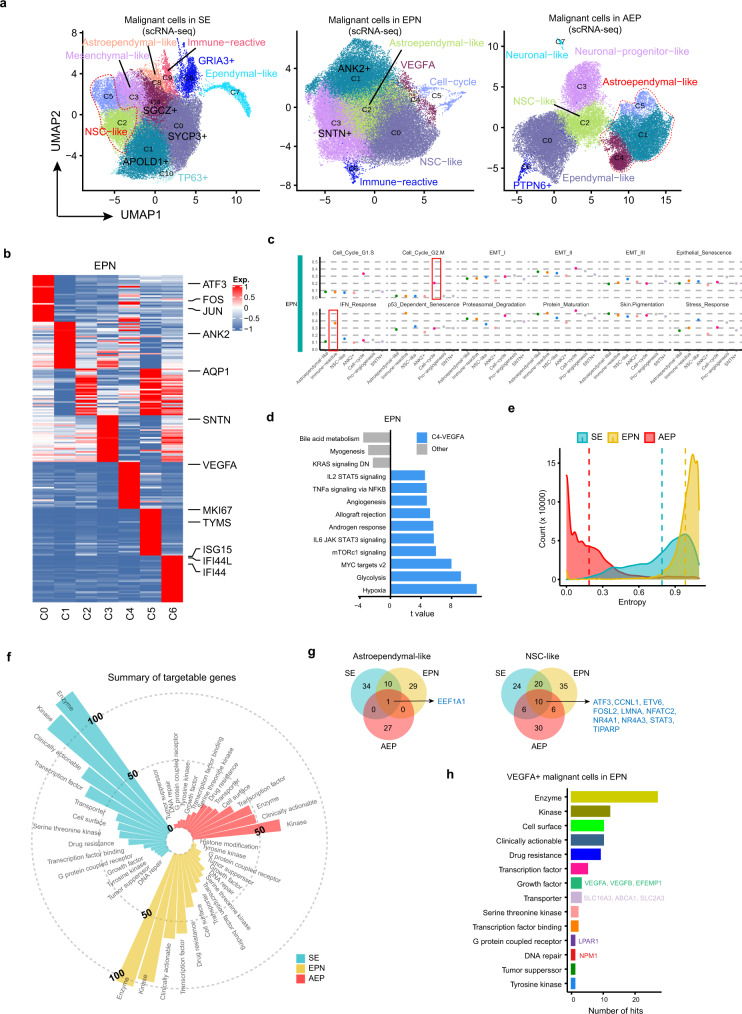
Heterogeneity of malignant cells in each ependymoma cancer subtype. **a** UMAP plots showing subclusters of malignant cells in each cancer subtype, donor effect corrected by Harmony. **b** Heatmap showing signature genes for each malignant cell subcluster in EPN. Selected genes were labeled on the right side. **c** Scatter plots showing average expression of 12 generic tumor cell programs in each malignant subset in EPN. The boxes highlight programs which were expressed in specific subsets. **d** Bar plot showing different pathways enriched in *VEGFA*^+^ C4 and other clusters from EPN scored per cell by gene set variation analysis (GSVA). *t* values were calculated with limma regression. **e** Density plot showing the entropy of patient distribution for malignant cells in each cancer subtype. **f** Circular bar plot showing the number of targetable genes from different categories in each cancer subtype. **g** Venn plots showing the intersection of targetable genes in astroependymal-like and NSC-like subpopulations across different cancer subtypes. The shared genes between the three cancer subtypes are listed on the right side. **h** Bar plot showing the number of targetable genes from different categories in *VEGFA*^+^ subpopulation of EPN. Source data are provided as a Source Data file. See also Supplementary Fig. 5 and Supplementary Data 6 and 7.

To study potential commonalities and correct for interindividual variations, we applied Harmony alignment^52^ to malignant cells in each cancer subtype separately. We identified multiple subclusters in each cancer subtype and assigned identities according to their signature genes (Fig. 4a, b, Supplementary Fig. 5a–c, and Supplementary Data 6). Using EPN as an example, our unsupervised clustering gave rise to seven subclusters. The first cluster, C0, was characterized by high expression of *ATF3*, *JUN*, and *FOS* and was termed NSC-like (neural stem cell-like) according to a previous study^19^. The C2 cluster exhibited high expression of *AQP1*, a marker of astrocytes, and was named astroependymal-like. The C5 cluster was characterized by specific expression of cell cycle genes, such as *MKI67* and *TYMS*, representing cells undergoing the cell cycle. The C6 cluster, termed immune-reactive, expressed high levels of interferon response genes, including *ISG15*, suggesting its potential roles in the immune response. The remaining subclusters were named according to their differentially expressed genes. Among the three subtypes, we identified both common (NSC-like and astroependymal-like) and cancer-specific subclusters, such as the immune-reactive subcluster in SE and EPN and the *VEGFA*^+^ subcluster in EPN (Fig. 4a). By evaluating the expression patterns of the 12 reported generic tumor cell programs^53^ across our malignant cells, we observed that the Cell_Cycle-related and IFN_Response programs were highly expressed in specific malignant subsets, but other programs were less different among malignant subsets (Fig. 4c and Supplementary Fig. 5d). Our analysis suggests that the generic tumor cell programs affected some of the malignant cells in our spinal ependymoma dataset.

Evaluating hallmark pathways in the *VEGFA*^+^ C4 cluster and other clusters from EPN by gene set variation analysis (GSVA) revealed a strong enrichment of angiogenesis and hypoxia in the *VEGFA*^+^ C4 cluster (Fig. 4d), suggesting its high capacity to favor tumor progression. We then compared cell fractions in each patient and observed that malignant cells from AEP displayed the highest patient specificity in cell-type composition (Supplementary Fig. 5e), which was further confirmed by our entropy analysis (Fig. 4e). We reasoned that this phenomenon might relate to the different cell types of origin in each AEP patient. Comparing malignant cells from our adult spinal ependymomas with those from pediatric brain ependymomas^19^, we observed that malignant cells isolated from spinal ependymomas in our study (from SE, EPN, and AEP) were grouped together, while malignant cells collected from pediatric brain ependymomas (from ST and PF) were grouped together, indicating that malignant cells from different anatomic locations exhibited diverse transcriptomic profiles (Supplementary Fig. 5f), highlighting the necessity of studying the transcriptomic characteristics of malignant cells in adult spinal cord ependymomas. These analyses described the heterogeneity of malignant cells and revealed different cellular compositions in each subtype.

To inform future therapeutic approaches targeting malignant cells of spinal ependymomas, we next examined potentially targetable biomarkers specific to the expression signatures of malignant cells in each cancer subtype. By comparing malignant cell population-specific genes with the Drug Gene Interaction database (DGIdb)^54^, we identified hundreds of pharmacologically targetable genes from different categories in each cancer subtype (Fig. 4f and Supplementary Data 7). Notably, for the common astroependymal-like and NSC-like subpopulations, although we revealed more cancer type-specific druggable vulnerabilities across cancer subtypes, the shared candidates, including the transcription factor gene *EEF1A1* and the kinase gene *CCNL1*, might help unify the pharmacological treatment of spinal ependymomas (Fig. 4g). For the *VEGFA*^+^ subpopulation in EPN, our analysis indicated druggable vulnerabilities, including the growth factor genes *VEGFA*, *VEGFB,* and *EFEMP1*, the transporter genes *SLC16A3*, *ABCA1*, and *SLC2A3*, the G protein-coupled receptor gene *LPAR1* and the DNA repair gene *NPM1* (Fig. 4h). We reasoned that these targetable genes revealed by our scRNA-seq data would provide valuable insights for future therapeutic strategies.

### Transcriptional differences reveal cancer subtype-specific characteristics

**Fig. 5:**
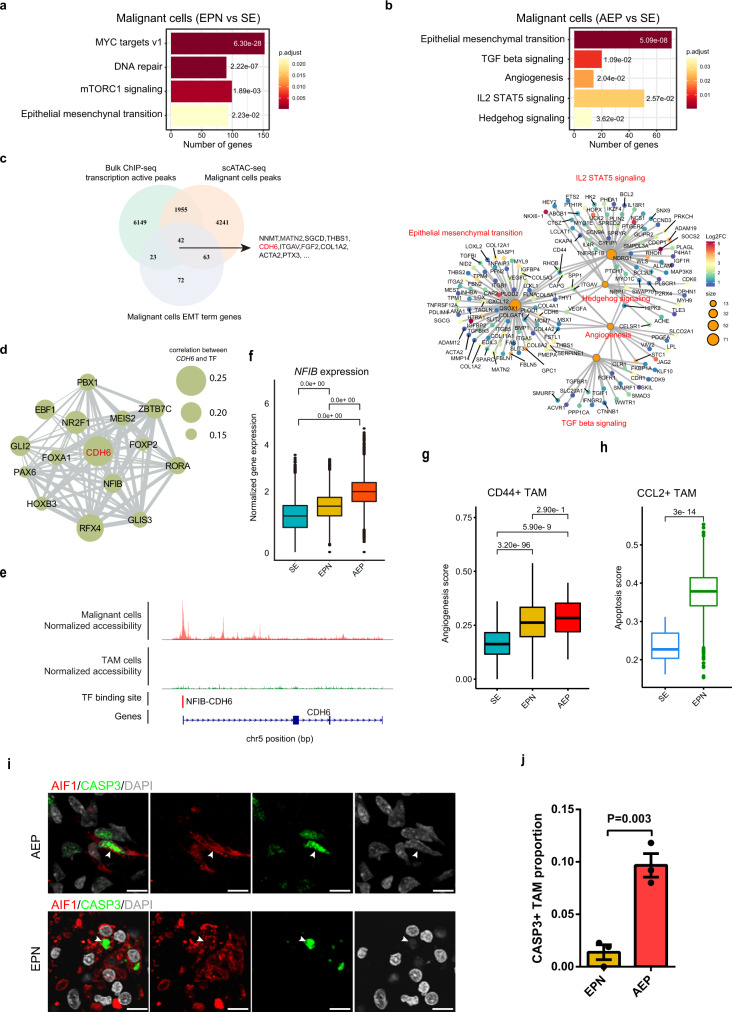
Transcriptional differences detected by scRNA-seq analyses reveal cancer subtype-specific characteristics. **a** Bar plot of enriched hallmark pathways for genes upregulated in malignant cells from EPN. Adjusted *P* values were labeled for each pathway. Adjusted *P* values were calculated by using *enrichr* function from R package clusterProfiler with hypergeometric test statistical analyses. **b** Bar plot of enriched hallmark pathways for genes upregulated in malignant cells from AEP (top). Network plot of enriched hallmark pathways for upregulated genes in malignant cells from AEP. Nodes for genes were colored by log2FC, and sizes of nodes for enriched pathways were correlated with the number of genes (bottom). Adjusted *P* values were labeled for each pathway. Adjusted *P* values were calculated by using *enrichr* function from R package clusterProfiler with hypergeometric test statistical analyses. **c** Venn plot showing the intersection of EMT-related genes with active transcriptional signals. The shared genes are listed on the right side. The red color marks the key genes of interest. **d** Network plot showing the connection between *CDH6* and its upstream TFs. Sizes of circles were related to the correlation value between *CDH6* and TFs. **e** Normalized scATAC-seq profile of *CDH6* in AEP across each major subpopulation and *NFIB-CDH6* binding site. **f** Box plot showing the normalized gene expression of *NFIB* from different cancer subtypes. Adjusted *P* values were calculated by the Wilcoxon test, two-sided comparisons. *n* = 122,456 cells. The center line, bounds of box, and whiskers represent mean, 25th to 75th percentile range, and minimum to maximum range in all boxplots. **g** Box plot showing the angiogenesis signature score of *CD44*^+^ TAMs from different cancer subtypes. Adjusted *P* values were calculated by the Wilcoxon test, two-sided comparisons. *n* = 3912 cells. The center line, bounds of box, and whiskers represent mean, 25th to 75th percentile range, and minimum to maximum range in all boxplots. **h** Box plot showing the apoptosis signature score of *CCL2*^+^ TAMs from different cancer subtypes. Adjusted *P* value was calculated by the Wilcoxon test, two-sided comparisons. *n* = 4647 cells. The center line, bounds of box, and whiskers represent mean, 25th to 75th percentile range, and minimum to maximum range in all boxplots. **i** Representative example of an EPN and AEP tumor stained by IF. The arrow depicts the CASP3^+^ TAMs in fluorescent images, and the scale bar represents 30 μm. **j** Bar plot showing the proportion of CASP3^+^ TAM in EPN and AEP (*n* = 3 biologically independent samples). The *P* value was calculated by *t* test, Two-way ANOVA analysis. Data are presented as mean values +/− SEM. Source data are provided as a Source Data file. See also Supplementary Fig. 5 and Supplementary Data 4 and 8–10.

We hypothesized that defining biological programs activated in cells from different cancer subtypes may explain their different degrees of malignancy. We first compared the transcriptomes of malignant cells from different cancer subtypes (Supplementary Data 8), and our enrichment analyses of hallmark pathways revealed specific pathways upregulated in tumor cells from AEP and EPN (Supplementary Data 4). Tumor cells from EPN exhibited an enrichment of the mTORC1 signaling pathway, while tumor cells from AEP showed a significant enrichment of the TGF-β, IL2/STAT5, and Hedgehog signaling pathways (Fig. 5a, b). In addition, tumor cells from AEP showed a significant enrichment of genes from angiogenesis pathways, including the genes *VEGFA* and *SPP1* (Fig. 5b), which play critical roles in controlling the growth of cancer by modulating blood supply in solid tumors^55^. Notably, one common upregulated pathway in AEP and EPN was the epithelial–mesenchymal transition (EMT) pathway (Fig. 5a, b), an important program that is involved in both cancer development and progression^56^. Using bulk histone modification ChIP-seq data and scATAC-seq data of malignant cells, we further investigated the epigenetic regulation of the EMT pathway in AEP. Since tumor cells occupied most of the tumor tissues, the bulk ChIP-seq data represented the characteristics of tumor cells. Therefore, we focused the multiomic analysis on only malignant cells and ultimately identified 42 EMT-related genes with active epigenetic signals (Fig. 5c and Supplementary Data 9). Gene *CDH6*, aberrantly reactivated in cancer and a TGF-β target that can drive the embryonic EMT pathway^57^, exhibited transcriptional activity around its TSS (Supplementary Fig. 5g) and was upregulated in AEP tumor cells (Fig. 5b). To further investigate the regulatory network associated with *CHD6*, we predicted its upstream TFs based on scATAC-seq data of tumor cells and revealed that *NFIB* was one of the upstream regulators (Fig. 5d, e). Moreover, we noticed that the expression of *NFIB* was upregulated in tumor cells from AEP (Fig. 5f), further implying that *NFIB* might have crucial effects on the enhanced activity of the EMT pathway in malignant cells.

We next examined the transcriptomic differences between TAM subsets in different cancer subtypes. Interestingly, we observed that *CD44*^+^ TAMs from EPN expressed higher levels of *SPP1* (Supplementary Data 10), which has been reported as a marker of angiogenesis-associated TAMs in colon cancer^18^. We further quantified the score of the angiogenesis pathway in *CD44*^+^ TAMs from each cancer subtype and observed significantly increased activity in *CD44*^+^ TAMs from EPN and AEP compared with that in *CD44*^+^ TAMs from SE (Fig. 5g), suggesting the stronger pro-tumorigenic properties of *CD44*^+^ TAMs in EPN and AEP. In addition, for *CCL2*^+^ TAMs, higher expression of *IL15*, which can reduce the immunosuppressive effects of regulatory T cells (Tregs)^58^, and lower expression of the immunosuppressive gene *TREM2*^59^ were observed in SE (Supplementary Fig. 5h), suggesting the higher antitumor capacity of *CCL2*^+^ TAMs in SE. In addition, we identified lower expression of the pro-apoptotic gene *PMAIP1*^60^ and higher expression of the anti-apoptotic gene *BCL2*^61^ in *CCL2*^+^ TAMs from SE (Supplementary Fig. 5h), indicating their lower apoptotic activity. Scoring the apoptosis signatures in *CCL2*^+^ TAMs in each cancer subtype further supported the notion that *CCL2*^+^ TAMs from EPN were more likely to undergo apoptosis (Fig. 5h). We confirmed that the proportion of CASP3^+^ TAMs was higher in AEP than in EPN by using IF staining (Fig. 5i, j), which complemented the limited number of *CCL2*^+^ TAMs in AEP found by our scRNA-seq data. Therefore, our analyses indicate that the *CD44*^+^ and *CCL2*^+^ TAMs, which played distinctive roles in inflammatory responses and regulating tumor growth, were correlated with the different degrees of malignancy across cancer subtypes, suggesting that the characteristics of TAMs could be used as a marker for different subtypes.

### Cell–cell interactions inform stromal cells to regulate TAM subset diversity

**Fig. 6:**
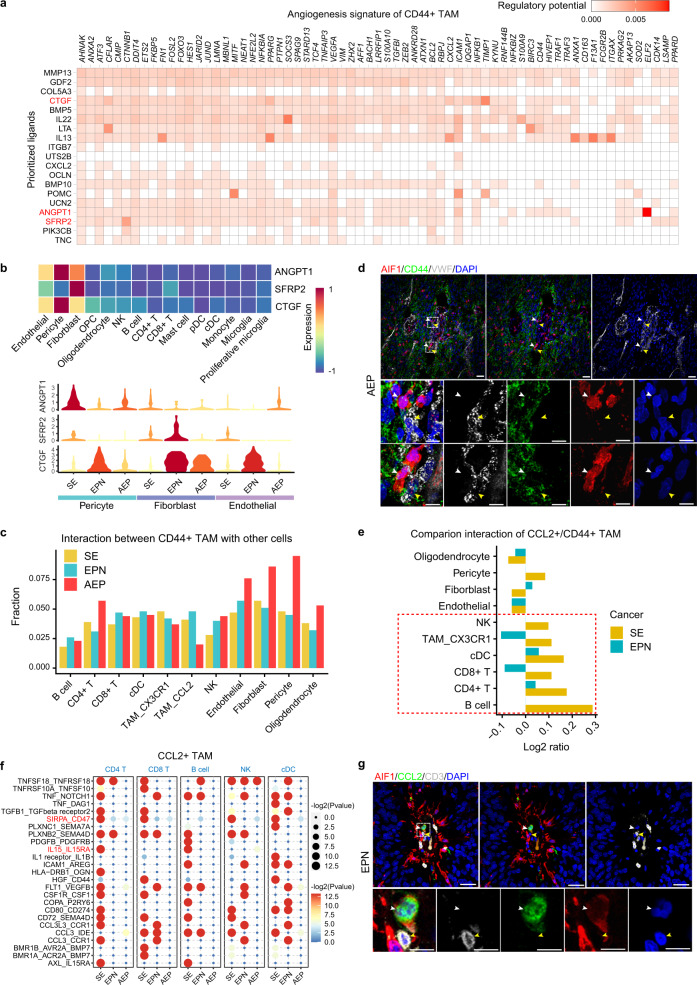
Cell–cell interaction analyses inform the mechanism of the formation of two TAM subsets. **a** Heatmap showing potential ligands driving the signature of *CD44*^+^ TAMs. The red color marks the genes of interest. **b** Heatmap showing the expression of selected ligands in stromal and immune cells (top). Violin plot showing the expression of selected ligands in fibroblasts, endothelial cells and pericytes from each cancer subtype (bottom). **c** Bar plot showing the fraction distribution of significant interaction events around *CD44*^+^ TAMs. The fraction of each interaction pair was calculated by dividing the total number of interaction events related to *CD44*^+^ TAMs. **d** Representative example of an AEP tumor stained by IF with anti-AIF1 (red), CD44 (green), VWF (gray), and DAPI (blue) antibodies. Dashed boxes highlight regions shown in the bottom panel. The white arrow depicts the *CD44*^+^ TAMs and the yellow arrow depicts the endothelial cells in fluorescent images. The scale bar in the top panel represents 30 μm, and the scale bar in the bottom panel represents 100 μm. Images shown are representatives of three samples from three independent experiments. **e** Bar plot showing the ratio of interaction events of *CCL2*^+^ TAMs to that of *CD44*^+^ TAMs. The fraction of each interaction pair was calculated by dividing the total number of interaction events related to *CD44*^+^ and *CCL2*^+^ TAMs. Source data are provided as a Source Data file. **f** Bubble heatmap showing selected significant LR pairs between *CCL2*^+^ TAMs and immune cells in each cancer subtype. Each row represents an LR pair, and each column defines a cell–cell interaction pair in a specific cancer subtype. *P* values were indicated by circle color and size. The red-color marking was the key ligand–receptor pair of interest. *P* values were calculated by CellPhoneDB. **g** Representative example of an EPN tumor stained by IF with anti-AIF1 (red), CCL2 (green), CD3 (gray), and DAPI (blue) antibodies. The white arrow depicts the *CCL2*^+^ TAMs and the yellow arrow depicts the T cells in fluorescent images. The scale bar in the top panel represents 30 μm and the scale bar in the bottom panel represents 100 μm. Images shown are representatives of three samples from three independent experiments. See also Supplementary Figs. 6 and 7.

To elucidate the underlying reasons for the functional diversity of TAM subsets, we first performed cell–cell interaction analysis by using CellPhoneDB^62^ (Supplementary Fig. 6a). Overall, tumor cells in each cancer subtype exhibited more interaction events with fibroblasts, endothelial cells and pericytes than with other cell types (Supplementary Fig. 6b). To explore extracellular signals that drive the special phenotype of *CD44*^+^ TAMs, we then performed NicheNet analysis^63^, which links ligand and target gene expression. Interestingly, NicheNet predicted a panel of ligands that might drive the unique signature genes of *CD44*^+^ TAMs (Fig. 6a), including three genes (*CTGF*, *SFRP2,* and *ANGPT1*) associated with angiogenesis^64–66^. Furthermore, we broadly surveyed the expression of the three ligands and observed that they were highly expressed in fibroblasts, endothelial cells, and pericytes, with diverse patterns across cancer subtypes (Fig. 6b). In addition, we observed more interaction events between *CD44*^+^ TAMs and fibroblasts and endothelial cells and pericytes in AEP (Fig. 6c), including the WNT5A-ROR2, JAG1-NOTCH3, and ANGPT2-TEK ligand–receptor (LR) pairs (Supplementary Fig. 6c), which are related to angiogenesis^67–69^. IF staining of EPN and AEP tumors also showed the physical juxtaposition of *CD44*^+^ TAMs and endothelial cells (Fig. 6d and Supplementary Fig. 6d). Our analysis thereby implied that communication with fibroblasts, endothelial cells, and pericytes might induce the angiogenesis phenotype of *CD44*^+^ TAMs.

We also performed NicheNet analysis to prioritize the ligands that induced the phenotype of *CCL2*^+^ TAMs and identified the apoptosis-associated ligand PGF^70^ (Supplementary Fig. 7a). Furthermore, we noticed that *PGF* was highly expressed in fibroblasts, endothelial cells and pericytes (Supplementary Fig. 7b), implying that these cells might be involved in inducing the apoptosis of *CCL2*^+^ TAMs. We next examined cell–cell interaction events around *CCL2*^+^ TAMs. Compared with *CD44*^+^ TAMs, *CCL2*^+^ TAMs showed more interactions with immune cells (Fig. 6e), consistent with their important roles in the inflammatory response. Close inspection of the LR pairs revealed significant enrichment of the IL15-IL15RA interaction between *CCL2*^+^ TAMs and *CD4*^+^ T cells in SE (Fig. 6f and Supplementary Fig. 7c), further supporting that the upregulation of *IL15* in SE might reduce the immunosuppressive effects of Tregs^58^. In addition, we identified significant interactions in SE between *CCL2*^+^ TAMs and other immune cells, including T cells, through SIRPA-CD47 (Fig. 6f), which might regulate their homeostasis and modulate their immune response^71^. We confirmed the physical juxtaposition of *CCL2*^+^ TAMs and T cells by IF staining (Fig. 6g and Supplementary Fig. 7d). These analyses informed us of a potential mechanism for the higher antitumor capacity of *CCL2*^+^ TAMs in SE.

## Discussion

Patients diagnosed with spinal ependymomas suffer from multiple symptoms, including neck and back pain, spasticity in the lower extremities, gait ataxia, sensory loss, paresthesias, and paralysis^4^. Due to the difficulty of surgical removal, new therapeutic strategies for spinal ependymomas are urgently needed clinically. Immunotherapy is believed to be the most promising approach for cancer treatment. Here, our analyses of scRNA-seq and scATAC-seq data characterized the cellular diversity in the TME of spinal ependymomas and highlighted two functionally diverse TAM subsets, providing insights for TAM-targeting immunotherapy.

*CCL2*^+^ TAMs, representing the activated TAMs in the TME, were more likely to interact with immune cells and showed a high immune-response capacity, implying their antitumor roles in ependymomas. We noticed that these *CCL2*^+^ TAMs exhibited high levels of apoptosis in EPN and AEP. Approaches that inhibit apoptosis of *CCL2*^+^ TAMs might help encourage the immune system to attack tumors. In contrast, *CD44*^+^ TAMs expressed high levels of angiogenesis-associated genes and tended to interact with endothelial cells, pericytes and fibroblasts to facilitate tumor angiogenesis. Here, we observed a higher pro-angiogenic capacity of *CD44*^+^ TAMs in EPN and AEP, providing a plausible explanation for the different degrees of malignancy across cancer subtypes. We reason that strategies that inhibit these *CD44*^+^ TAM populations might decelerate the progression of ependymomas. Numerous studies have shown the therapeutic targeting of TAMs can contribute to tumor immunotherapy and the strategies can be roughly divided into three approaches^72^: eliminating TAMs already present in the TME^73,74^, inhibition of monocyte recruitment^75^ and reprogramming of TAMs^76^. Clodronate-loaded and zoledronate acid-loaded liposomes have been applied to deplete TAMs resulted in decreasing tumor angiogenesis and progression in preclinical solid tumor models successfully, which indicate that these treatments could also be used to deplete *CD44*^+^ TAMs in spinal ependymoma.

To investigate the intracellular factors that mediated the functional diversity of TAMs, we further performed scATAC-seq data analysis and revealed that TEAD1 and EGR3 regulated the functional diversity of the *CD44*^*+*^ and *CCL2*^*+*^ TAM subsets, respectively. Previous studies reported that TFs from the TEAD family have a substantial effect on cancer development, progression, and metastasis, suggesting that TEAD proteins could serve as potential therapeutic targets^77–79^. Here, we discovered the regulatory roles of TEAD1 in *CD44*^*+*^ TAMs, further highlighting its potential application in ependymoma immunotherapy. Moreover, EGR3 motif enrichment analysis showed the strong correlation of EGR3 with the TNF pathway in *CCL2*^+^ TAMs; this result indicates that EGR3 plays important role in tumor inflammation, which was consistent with the tumor-suppressive role of EGR3 in certain cancer events^80^.

For intercellular factors, we uncovered the roles of stromal cells, including fibroblasts, endothelial cells, and pericytes in regulating the functional diversity of TAM subsets in spinal ependymomas. Furthermore, we prioritized multiple ligands that might induce the phenotypes of the *CD44*^+^ and *CCL2*^+^ TAM subsets. For example, we predicted the regulatory relationship between the ligand PGF and apoptosis-associated genes (*NEDD9* and *PMAIP1*) in *CCL2*^+^ TAMs, implying that factors blocking PGF might inhibit the apoptosis of *CCL2*^+^ TAMs. These findings invite more investigations to reveal the molecular mechanisms underlying the phenotypic diversity of TAMs, which would further facilitate the development of TAM-targeting immunotherapy.

By comparing malignant cells from our adult spinal ependymoma with those from pediatric brain ependymoma with single-cell transcriptomic analysis, we found that the malignant cells of ependymomas exhibited heterogeneity across anatomic locations, suggesting that knowledge of one type of ependymoma may be unable to be applied to others. By analyzing TAMs isolated from other CNS gliomas, we observed that *CD44*^*+*^ TAMs were also present in GBM, showing a similar expression pattern as those in ependymoma, although they hardly existed in gliomas, indicating that TAM-targeting immunotherapy strategies based on these dual-function TAMs are applicable to multiple types of tumors but not all neurological tumors.

From this study, we were able to summarize potentially targetable biomarkers in malignant cells from each spinal ependymoma subtype, which could inform future therapeutic approaches targeting spinal ependymomas. We also identified diverse activity of the EMT pathway across different spinal ependymoma subtypes, which was associated with their increasing degrees of malignancy. Furthermore, by combining the results of scRNA-seq, scATAC-seq, and bulk ChIP-seq data analyses, we identified the NFIB-CDH6-regulatory program as a potential mediator of the enhanced activity of the EMT pathway.

Overall, our multiomics analyses characterized spinal ependymomas heterogeneity at both the transcriptomic and epigenomic levels. In particular, we highlighted the multifaceted roles of TAMs in tumor progression, indicating that TAM subtype-specific gene expression could be an indicator of the degree of malignancy and that TAM subtype-targeting immunotherapy may have the potential to enhance the immune system to eliminate tumors.

## Methods

### Patient samples

Fifteen treatment-naïve patients who were pathologically diagnosed with ependymoma subtypes, including seven males and eight females, were enrolled in this study after approval by the Ethics Committee of Beijing Tiantan Hospital, Capital Medical University. All patients provided written informed consent for sample collection and data analyses. We summarized the available clinical characteristics in Supplementary Data 1.

### Tumor tissue harvest and dissociation for single-cell suspension

#### Live-cell isolation from fresh tissue

We collected fresh tumor tissue during surgery and stored in cold phosphate-buffered saline (PBS) to transfer to the laboratory on ice immediately. Tumor tissue was dissociated mechanically with the digestion buffer (2 mg/ml collagenase IV (Gibco), 10 U/ml DNase I (NEB), and 1 mg/ml papain (Sigma) in PBS). After incubation for about 20–30 min at 37 °C on a thermocycler, 10% fetal bovine serum (Gibco) was used to stop the digestion system. Single-cell suspensions dissociated from the tumor were resuspended in 0.04% BSA/PBS and stained with 7-amino-actinomycin D (7-AAD) to sort 7-AAD-negative cells by FACS for single-cell libraries preparation.

#### Nuclei isolation from frozen tissue

Frozen tumor tissue was homogenized into small cell pellets using a glass tissue grinder (Sigma, Cat #D8938) in ice-cold EZ buffer (Sigma, Cat #NUC-101) and incubated on ice for 5 min. Then nuclei were centrifuged at 500 × *g* for 5 min at 4 °C. Repeat the wash and centrifuge step again. After that, nuclei were resuspended with PBS, and Debris Removal Solution (MACS) was added to effectively remove cell debris. Isolated nuclei were suspended in Nuclei Suspension Buffer (NSB; consisting of 1× PBS, 0.01% BSA and 0.1% RNase inhibitor (Clontech, Cat #2313A)). A final nuclei suspension was filtered through a 35-μm cell strainer (Corning, Cat #352235) and used for single-cell libraries preparation.

#### Library preparation and sequencing

Single-cell/nuclei were processed using Chromium Single Cell V2 or V3 Chemistry Library Kits (10× Genomics) and GEM ATAC v1.1 Kits (10× Genomics) according to the manufacturer’s instructions. Thousands of cells/nuclei were partitioned into Gel Beads-in-emulsion (GEMs) on the Chromium Controller, followed by full-length cDNA generated, barcoded, and sequenced on an Illumina NextSeq4000.

### Immunohistochemistry

We dropped fresh tumor tissue in 4% paraformaldehyde overnight at 4 °C. After being washed with PBS three times for 10 min, the fixed tissue was placed in 20% and 30% sucrose solution to dehydrate and then was embedded in optimal cutting temperature medium (Thermo Scientific). Embedded tissue was sectioned at 20–30 μm cryosections using Leica CM3050S cryostat. Before IHC staining, tissue slices were subjected to heat-mediated antigen retrieval with Tris/EDTA buffer (PH 9.0). Then, tissue slices were blocked in 5% donkey serum for 2 h. Sections were incubated with primary antibody at the following dilutions: goat anti-IBA1 (1:100, Abcam, ab5076), Rabbit anti-MCP1 (1:100, Abcam, ab73680), mouse anti-CD44 (1:100, Abcam, ab16728), rabbit anti-VWF (1:100, Abcam, ab9378), mouse anti-CD3 (1:50, Abcam, ab699), rabbit anti-cleaved Caspase-3 (1:200, Cell Signaling, #9661), rabbit anti-TMEM119 (1:1000, Abcam, ab185333). After incubation overnight at 4 °C with primary antibody, Alexa Fluor 488, 594, or 647 fluorophore-conjugated secondary antibodies (1:500) (Life Technologies), and DAPI (1:500) were added to slices incubation buffer for 2 h. We used Olympus FV3000 confocal microscope for collecting images.

### Chromatin immunoprecipitation-sequencing (ChIP-seq)

CUT& Tag ChIP-seq were performed as described^81^. Nuclei were washed twice in Wash Buffer (20 mM HEPES pH 7.5; 150 mM Nacl; 0.5 mM Spermidine; 1× Protease inhibitor cocktail). Add 10 μl Concanavalin A-coated magnetic beads (Bangs Laboratories) per sample and incubated at RT for 15 min. Remove supernatant and resuspend in Dig-wash Buffer (20 mM HEPES pH 7.5; 150 mM NaCl; 0.5 mM Spermidine; 1× protease inhibitor cocktail; 0.05% digitonin), supplementing 2 mM EDTA and the primary antibody in 1:50 dilution. Incubate primary antibody overnight at 4 °C and then replaced it with secondary antibody (1:50 diluted in Dig-Wash buffer). Secondary antibody incubation was performed at room temperature (RT) for 30 min. Unbound antibodies in Nuclei was washed using the magnet stand 2–3 times for 5 min. pA-Tn5 adapter complex was diluted in 1:200 using Dig-med Buffer (0.05% digitonin, 20 mM HEPES, pH 7.5, 300 mM NaCl, 0.5 mM Spermidine, 1× protease inhibitor cocktail). Nuclei was resuspended in Dig-med Buffer and incubated with pA-Tn5 at RT for 1 h. After washed, nuclei were resuspended in Tagmentation buffer (10 mM MgCl_2_ in Dig-med Buffer) and incubated at 37 °C for 1 h. 2.25 µL of 0.5 M EDTA, 2.75 µL of 10% SDS, and 0.5 µL of 20 mg/mL Proteinase K were added to 50 µL of sample to stop tagementation and then inactivate Proteinase K at 70 °C for 20 min. Ampure XP beads were used to extract DNA. DNA was amplified using universal i5 and uniquely barcoded i7 primer and cleaned up by adding 1.1× Ampure XP beads (Beckman Colter). DNA libraries were sequenced on an Illumina NextSeq4000.

### scRNA-seq data processing

scRNA-seq experiments were performed on 15 samples (Supplementary Data 1), the raw data of which are accessible under database Genome Sequence Archive (GSA: HRA001112) or database Gene Expression Omnibus (GEO: GSE163686). scRNA-seq data generated from 10× Genomics were processed using the Cell Ranger Single-Cell Software Suite (v3.0.1), which aligned sequencing reads to hg19 human reference genome and quantified gene expression levels of single cells. We merged cells from different tumors using the *CellRanger aggr* pipeline and the preliminary filtered data were used for downstream analysis. First, we applied further quality control to cells according to three different metrics step by step, including the total UMI count, the number of detected genes, and the proportion of mitochondrial gene count per cell. Specifically, we filtered cells with less than 2000 UMI count or 500 detected genes, as well as cells with more than 20% mitochondrial gene count. To remove potential doublets, we also removed cells with UMI count above 70,000 and the number of detected genes above 10,000. Here, we used a relatively high threshold because malignant cells are thought to express a greater number of genes. Furthermore, we removed potential doublets predicted by Scrublet^82^. Next, after quality control, we applied the library-size correction method to normalize the raw count matrix by using *normalize_total* function in SCANPY^83^. Then the logarithmized normalized count matrix was used for the downstream analysis.

### Dimension reduction and unsupervised clustering

We employed the workflow of SCANPY^83^ to perform dimension reduction and graph-based unsupervised clustering on scRNA-seq data. Briefly, we first selected 2000 highly variable genes (HVGs) for downstream analysis by using *scanpy.pp.highly_variable_genes* function with parameter “*n_top_genes=2000*”. Then, effects of the total count per cell, the percentage of mitochondrial gene count and the percentage of count for heat shock protein associated genes (HSP) were regressed out by using *scanpy.pp.regress_out* function. Principal component analysis (PCA) was performed on selected HVGs by using *scanpy.tl.pca* function with parameter “*svd_solver='arpack', n_comps=100*”, and generated a PCA matrix with 100 components. Next, we employed *bbknn* algorithm with parameter “*batch_key='patient', n_pcs=90*” to remove the batch effects from different donors and obtain a batch-corrected space. To visualize our scRNA-seq data in a two-dimensional space, Uniform Manifold Approximation and Projection (UMAP) dimension reduction was performed by using *scanpy.tl.umap* function with default parameter. Last, to cluster single cells by their transcriptional profiles, we used an unsupervised graph-based clustering algorithm implemented in *scanpy.tl.leiden* function with different *resolution* parameters adapted to diverse situations. The cluster-specific marker genes were identified by using *scanpy.tl.rank*_genes_groups function with default parameter. Specifically, we performed two round of dimension reduction and unsupervised clustering for all cells. The first round (“*resolution = 2*”) was used to distinguish malignant cells from nonmalignant cells and the second round (“*resolution = 1*”) was applied to nonmalignant and malignant cells respectively. Notably, we used *scanpy.pp.neighbors* function with default parameter to compute a neighborhood graph instead of *bbknn* to avoid eliminating the great donor effects in malignant cells. Subclusters within nonmalignant cells were annotated by using canonical markers shown in Fig. 1e. To reveal the cellular diversity in monocytes and TAM, we re-run the dimension reduction and unsupervised clustering with “*resolution = 0.6*”.

### InferCNV analysis

Copy-number variations (CNVs) of cells were estimated by computing a moving average of the relative expression by using InferCNV (inferCNV of the Trinity CTAT Project, https://github.com/broadinstitute/inferCNV)^84^. Nonmalignant cells including both stomal and immune cells were used as to define a baseline of normal karyotype and their average copy-number value was subtracted from all cells.

### Enrichment analysis

We used *enrichr* and *GESA* functions from R package clusterProfiler for hypergeometric test and gene set enrichment analysis, respectively. Network plots of enriched pathways were visualized by function *cnetplot* from R package enrichplot. The R package GSVA from Bioconductor was used for gene set variation analysis. The gene sets (MSigDB) was loaded from R package msigdf and pathways with high difference in activity scores were selected by LIMMA package.

### Survival analysis

We used the top 30 genes of *CD44*^+^ TAM in Supplementary Data 3 to perform survival analysis by using the GEPIA2 webserver (http://gepia2.cancer-pku.cn/). Two cancer types (LGG and GBM) were selected to test the correlation between the given signature gene list and patients’ survival time with default parameters.

### Quantification of gene set score

We used the average expression level of a given gene set to quantify gene set score in every single cell. The M1 and M2 signatures were extracted from the previous publication^13^. Other gene sets, including angiogenesis and apoptosis were loaded from R package msigdf.

### SCENIC analysis

We used SCENIC^34^ with raw count matrices as input to analyze activated regulons in each TAM subset. Briefly, we inferred the coexpression network by using *GRNBoost2* python package and identified regulons by using *RcisTarget* package. Then, regulon activity for each cell was quantified by *AUCell* package, and differentially activated regulons in each TAM subset were identified by *wilcox.test* using other subsets as control. Finally, Benjamini–Hochberg procedure was used to correct the multiple hypothesis.

### Developmental trajectory analysis

#### RNA velocity

We used Velocyto pipeline^85^ to annotate spliced and unspliced reads and generated the loom file for downstream analysis. Then the python package scVelo^86^ was used to estimate the steady-state RNA velocities by using default parameters. Finally, the velocities were projected onto UMAP embedding and visualized on the cellular level by using *scv.pl.velocity_embedding* function. Here we removed *CD16*^*+*^ monocytes because monocyte-derived macrophages were more likely to develop from *CD14*^*+*^ monocytes^18^.

#### PAGA

We further used the partition-based graph abstraction method PAGA^83^ to assess the developmental trajectories for TAMs. The computations were carried out using default parameters. The edge connectivity with *CD14*^*+*^ monocytes between each subpopulation node are further visualized in the bar plot.

#### Predicting the origins of TAMs by using SingleR

To quantify the fraction of TAMs from different origins (microglia or monocytes), we used SingleR^87^, a predictor mainly based on transcriptomic similarity with the reference dataset. Here, we used the published data^47^ with both Mg-TAMs (microglia-derived TAMs) and Mo-TAMs (monocyte-derived TAMs) as a reference.

### Integration of TAMs in our study with that from other neurological tumors

We used Harmony^52^ to integrate TAMs from different studies. Briefly, TAMs were first performed PCA analysis with 100 components by using the top 2000 highly variable genes identified by *scanpy.pp.highly_variable_genes* function with parameter “*n_top_genes=2000*”. Then Harmony was applied to the 100 principle components with default parameters to integrate TAMs from different studies and generate a corrected embedding. Next, we used the first 30 Harmony corrected dimensions to compute a neighborhood graph by using *scanpy.pp.neighbors* function with default parameter. Finally, the UMAP dimension reduction was then performed by using *scanpy.tl.umap* function with the default parameter.

### Data harmonization and unsupervised clustering for malignant cells

We applied Harmony alignment^52^ to malignant cells from different patients to study their potential commonalities. We adopted two different algorithms to correct different sources of intersample variations. BBKNN uses the mutual nearest neighbors (MNNs) between different batches to correct batch effects, while Harmony deploys a local correction idea that preferentially clusters cells from different batches, thereby better matching the distributions of the shared cell types across donors. For each cancer type, malignant cells were first performed PCA analysis with 100 components as described earlier. Harmony was then applied to the 100 principle components with default parameters to integrate malignant cells from multiple patients and generate a corrected embedding. Next, we used the first 20 Harmony corrected dimensions to compute a neighborhood graph by using *scanpy.pp.neighbors* function with parameter “*n_neighbors=10*”. Uniform Manifold Approximation and Projection (UMAP) dimension reduction was then performed by using *scanpy.tl.umap* function with default parameter. Last, we used the same unsupervised graph-based clustering algorithm to cluster malignant cells and the same function for marker gene identification as described earlier. Notably, we excluded three patients (EPN2, EPN3, and EPN5) in this analysis because of the limited malignant cell numbers.

### Evaluation of mixability with entropy metric

We adopted an entropy-based metric to quantify the mixability of malignant cells across different patients after data harmonization^13^. Specifically, we randomly sampled 25,000 malignant cells and constructed a kNN graph (k = 80) based on the Euclidean distance in UMAP coordinates by using function *kNN* from R package *dbscan*. We defined the mixability of cells across patients as Shannon entropy,

1 $${H}_{i}=-\mathop{\sum }\limits_{d=1}^{D}{p}_{i}^{d}{{{\log }}}_{2}{p}_{i}^{d}$$

where ${p}_{i}^{d}$ is the fraction of cells from patient *d* in the 80 nearest neighborhoods of cell *i* and ${\sum }_{d=1}^{D}{p}_{i}^{d}=1$.

### Targetable biomarkers identification

Drug Gene Interaction database (DGIdb)^54^, a valuable resource that focuses on expert-curated collections of druggable genes, was used to examine potential targetale biomarkers within the specific gene signatures (top 100 genes reported by *scanpy.tl.rank*_genes_groups function) of each malignant cell subpopulation from each cancer subtype. We excluded DGIdb hits that were categorized as druggable genome, which was predicted on the basis of sequence and structural similarity^88^.

### Differential analysis for malignant cells from different cancer types

Here we first combined the raw count matrix of malignant cells from each cancer type together and then used the *sc.pp.normalize_total* function from SCANPY to normalize the combined data matrix (library-size corrected), which made the counts comparable among cells. Finally, the logarithmized count matrix was used for differential analysis by the SCANPY function *sc.tl.rank_genes_groups* using the Wilcoxon test.

### Comparison of adult spinal cord ependymoma and pediatric brain ependymoma

To compare malignant cells from our study with that from a published dataset, we first extracted the top 50 signature genes (reported by SCANPY) for each malignant cell subpopulation and then aggregated their averaged expression level in each cell population within each tumor type. Finally, the pairwise correlation of the averaged expression level was calculated by *cor* function in R with default parameters and visualized in the heatmap.

### Cell–cell interaction analysis

We applied CellPhoneDB^62^ to infer cell–cell interaction between cells in each cancer type. The enriched ligand–receptor interactions between two cell subsets were calculated based on the permutation test. We extracted significant ligand–receptor pairs with *P* value < 0.05, and summarized the number of interaction events related with tumor cells by the union of significant ligand–receptor pairs. Circle plots depicting the number of interactions between cell types are drawn using R package circlize.

### NicheNet analysis

We used NicheNet^63^ to predict ligands that drive the signature genes of the two TAM subsets. We first calculated DEGs in both *CCL2*^+^ and *CD44*^+^ TAMs. Then, DEGs with log2FC > 0.2 and adjusted *P* value < 0.05 in each TAM subset were used as gene sets of interest, respectively. All expressed genes in *CCL2*^+^ or *CD44*^+^ TAMs were used as the background of genes. Genes were considered as expressed when they have nonzero values in at least 10% of the cells in a cell type. Here, sender cell type was not specified to broadly examine ligand’s activity.

### Evaluating expression patterns of the reported generic tumor cell programs across malignant cell subsets

We downloaded the 12 generic tumor cell programs from the pan-cancer single-cell analysis of cancer cell lines^53^. The average expression of each generic tumor cell program in each malignant cell was calculated and scaled to 0–1. To quantify the aggregated expression level of each program in each malignant subset, we further calculated the average expression scores for each program across each subset and visualized their patterns in scatter plots.

### Data processing of single-cell ATAC-seq

scATAC-seq experiments were performed on two samples (Supplementary Data 1), the raw data of which are accessible under database Genome Sequence Archive (GSA: HRA001112) with GSA individual accession number HRI137901 and HRI137902. scATAC-seq raw data for samples HRI137901 and HRI137902 can be obtained under GSA Run accession HRR337425 and HRR337426 respectively. The Cell Ranger software (v3.0.1)^89^ performed reads filtering, alignment, and transposase cut sites identification. Cell by feature matrix with a window size of 2.5 kb was generated as described previously^89^ by first reading fragment file into R. Next, the GenomicRanges of the fragments were concatenated for each “start” and “end” followed by identifying all overlaps with the feature by insertions using R function *findOverlaps*. Then the fraction of Tn5 insertions was computed by dividing the cells-wise sum of the count matrix by the number of insertions for each cell. The processed count matrix was first binarized and then log-normalized for downstream analysis. The binarized count matrix was passed to Signac^90^ for dimension reduction and clustering. Signac function *RunTFIDF* and *RunSVD* were performed with default parameters for dimension reduction analysis. Briefly, the count matrix was the first frequency-inverse document frequency (TF-IDF) transformed by dividing each index by the cellwise sum of the matrix multiplying with the inverse document frequency computed as log(1 + ncol(matrix)/rowSums(matrix)). Singular value decomposition (SVD) was then performed on the TD-IDF matrix for dimension reduction. Clustering was done with the function *FindNeighbors* and *FindClusters*. Peak calling for each cluster was performed with fragments from cells in the cluster using MACS2^91^ with parameters ‘–nomodel –shift 75 –extsize 150 –qval 5e-2 -B -f BED –nolambda –keep-dup all –call-summits’ callpeak with MACS2 software (v 2.2.7.1). We retained those peaks only whose *q* values were less than 0.05 for further analysis and considered these peaks as reproducible peaks. The *q* value of each peak call is computed adjusting *P* value to control the false discovery rate with multiple testing correction which is a measure of statistical significance of the peak call. Peaks from each cluster were then merged and a binarized cell-by-peak matrix was constructed by converting nonzero counts to 1. The generated cell-by-peak matrix was then used to create a Signac object. Cells that pass the following quality criterion were kept: (1) the number of fragments in peaks between 1000 and 20,000; (2) the fraction of fragments in peaks greater than 10%; (3) transcriptional start site (TSS) enrichment score greater than 2; (4) ratio reads in genomic blacklist regions smaller than 0.05; (5) nucleosomal signal strength smaller than 4. In total, 4922 cells from two samples were initially profiled, and 2845 cells passed the quality control for further analyses. The filtered cell-by-peak matrix was passed to Signac function *RunTFIDF* and *RunSVD* for dimension reduction and function *FindNeighbors* and *FindClusters* for cluster identification. *FindNeighbors* was performed with reduced dimension components 2:30. *FindClusters* was performed by setting resolution to 0.3. Next, gene activity matrix was generated by quantifying the activity of each gene in the genome by accessing gene-associated chromatin accessibility using function *GeneActivity*. Clusters were then annotated based on gene activity score profiles of known cell-type markers. *TEKT1*, *GFAP*, *OLIG2*, *NEUROD6*, and *CD68* were used to name major cell types as ependymal-like cells, astrocytependymal-like cells, oligodendrocyte progenitor cell (OPC), neuronal-like cells, and TAMs, respectively.

### Calculation of differentially accessible peaks

Differentially accessible peaks were computed with Signac function *FindAllMarkers* by setting parameters *test.use =* “*LR*” and *latend.vars =* “*peak_region_fragments*”. Only peaks with adjusted *P* value smaller than 0.05 were considered as differentially accessible peaks.

### Co-accessible regulatory network

To build co-accessible regulatory network, we first identified upstream transcription factors of *CDH6* whose peaks were enriched for the malignant cells. Briefly, we computed differentially accessible peaks among the malignant and microglia clusters with Signac function *FindAllMarkers*, followed by identifying closest genes to each of these peaks with function *ClosestFeature*. We then performed motif analysis for searching transcription factor motifs closed to the transcription start site (TSS) of *CDH6*. Next, we computed the intersection of the upstream TFs of *CDH6* and the closest genes around those peaks that were more accessible for malignant cells. Then, we computed correlation of the transposed gene activity score matrix (cell in row and gene in the column) containing only *CDH6* and those intersected TFs. The correlation matrix was then loaded into software Cytoscape^92^ for network visualization. Edges with a correlation coefficient smaller than 0.15 were removed. The width of the edges and the size of nodes were scaled with the correlation coefficients.

### Integration of scRNA-seq and scATAC-seq data

To integrate scATAC-seq data with snRNA-seq data, we first identified anchors between these two datasets with Signac function *FindTransferAnchors* based on the expression and gene activity profiles of highly variable genes identified from the scRNA-seq data. We then computed the imputed gene expression for scATAC-seq cells based on the previously computed anchors with function *TransferData*. To construct co-embedding for the scRNA-seq and scATAC-seq data, we next run Signac function *RunPCA* and *RunUMAP* on the imputed gene expression matrix.

### Motif enrichment

Motif enrichment was performed with FIMO from the MEME suites^93^ to identify enriched binding motif in the genes’ regulatory regions. Homer^94^ function *annotatePeaks.pl* was then used for motif binding site annotation.

### Visualizing differentially accessible peaks

The deeptools^95^ was applied to visualize pileups of cluster-specific ATAC-seq signals in differentially accessible peaks.

### ChIP-seq data processing

ChIP-seq experiments were performed on two samples (Supplementary Data 1), the raw data of which are accessible under database Genome Sequence Archive (GSA: HRA001112) with GSA individual accession number HRI137901 and HRI137902. H3K27ac and H3K4me3 ChIP-seq data for sample HRI137901 can be obtained under GSA Run accession HRR337427 and HRR337429 respectively. H3K27ac and H3K4me3 ChIP-seq data for sample HRI137902 can be obtained under GSA Run accession HRR337428 and HRR337430 respectively. Fastq-files were trimmed for adaptor sequence using cutadapt (3.2)^96^ and then mapped to hg19 using Bowtie2 (2.4.2)^97^ with parameters “*-3 3 –no-discordant –no-mixed*”. SAMtools (1.9) and bedtools (2.30.0) were applied to filter mapped read pairs post alignment. MACS2 (2.2.7.1) was used for peak calling with default parameters^98^. The visualization of peaks was realized by IGV. For each sample, we identified active promoters by H3K4me3 peaks that overlap H3K27ac within 1000bp to the nearest transcription start site (TSS).

### Reporting summary

Further information on research design is available in the Nature Research Reporting Summary linked to this article.

## Supplementary information

Supplementary Information

Description of Additional Supplementary Files

Supplementary Data 1

Supplementary Data 2

Supplementary Data 3

Supplementary Data 4

Supplementary Data 5

Supplementary Data 6

Supplementary Data 7

Supplementary Data 8

Supplementary Data 9

Supplementary Data 10

Reporting Summary

## Data Availability

The scRNA-seq data generated in this study are available in the Gene Expression Omnibus (GEO) database under accession number GSE163686 and also available in the Genome Sequence Archive (GSA) database under accession number HRA001112. The scATAC-seq and ChIP-seq data generated from two samples in this study are available in the GSA database under accession number HRA001112. In particular, H3K27ac and H3K4me3 ChIP-seq data for sample HRI137901 can be obtained under GSA accession numbers HRR337427 and HRR337429, respectively. H3K27ac and H3K4me3 ChIP-seq data for sample HRI137902 can be obtained under GSA accession numbers HRR337428 and HRR337430, respectively. scATAC-seq data for samples HRI137901 and HRI137902 can be obtained under accession numbers HRR337425 and HRR337426, respectively. The data in GSA are available under restricted access, access can be obtained by contacting Xiaoqun Wang (xiaoqunwang@ibp.ac.cn). The published datasets used in this study can be downloaded from https://www.brainimmuneatlas.org/ or from GEO under accession number GSE163120^47^; scRNA-seq data for TAMs from glioblastoma, GSE166418^51^; scRNA-seq data for TAMs from IDH-mutant high-grade gliomas, GSE84465^50^; scRNA-seq data for TAMs from glioblastoma and GSE67835^49^; scRNA-seq data for macrophage from normal brain. The remaining data are available within the Article, Supplementary Information or Source Data file. Source data are provided with this paper.

## Source data

Source Data
