# Supplementary material for: Interrogation of the microenvironmental landscape in spinal ependymomas reveals dual functions of tumor-associated macrophages
Source: Nat Commun. 2021 Nov 25;12:6867. doi: 10.1038/s41467-021-27018-9 (PMC8617028; doi:10.1038/s41467-021-27018-9)
Supplement: Supplementary file 1 — Supplementary Information [file 41467_2021_27018_MOESM1_ESM.pdf]

Supplementary information

Interrogation of the microenvironmental landscape in spinal ependymomas reveals dual functions of tumor-associated macrophages

Qianqian Zhang[1,2,#], Sijin Cheng[1,#], Yongzhi Wang[3,4, #], Mengdi Wang[1,#], Yufeng Lu[1,2], Zengqi Wen[1], Yuxin Ge[5], Qiang Ma[1,2], Youqiao Chen[5], Yaowu Zhang[3,4], Ren Cao[3,4], Min Li[1,4], Weihao Liu[3,4], Bo Wang[3,4], Qian Wu[5,6*], Wenqing Jia[3,4,*], Xiaoqun Wang[1,2,6,7,8,*]

Contents:

Supplementary Figures 1-7

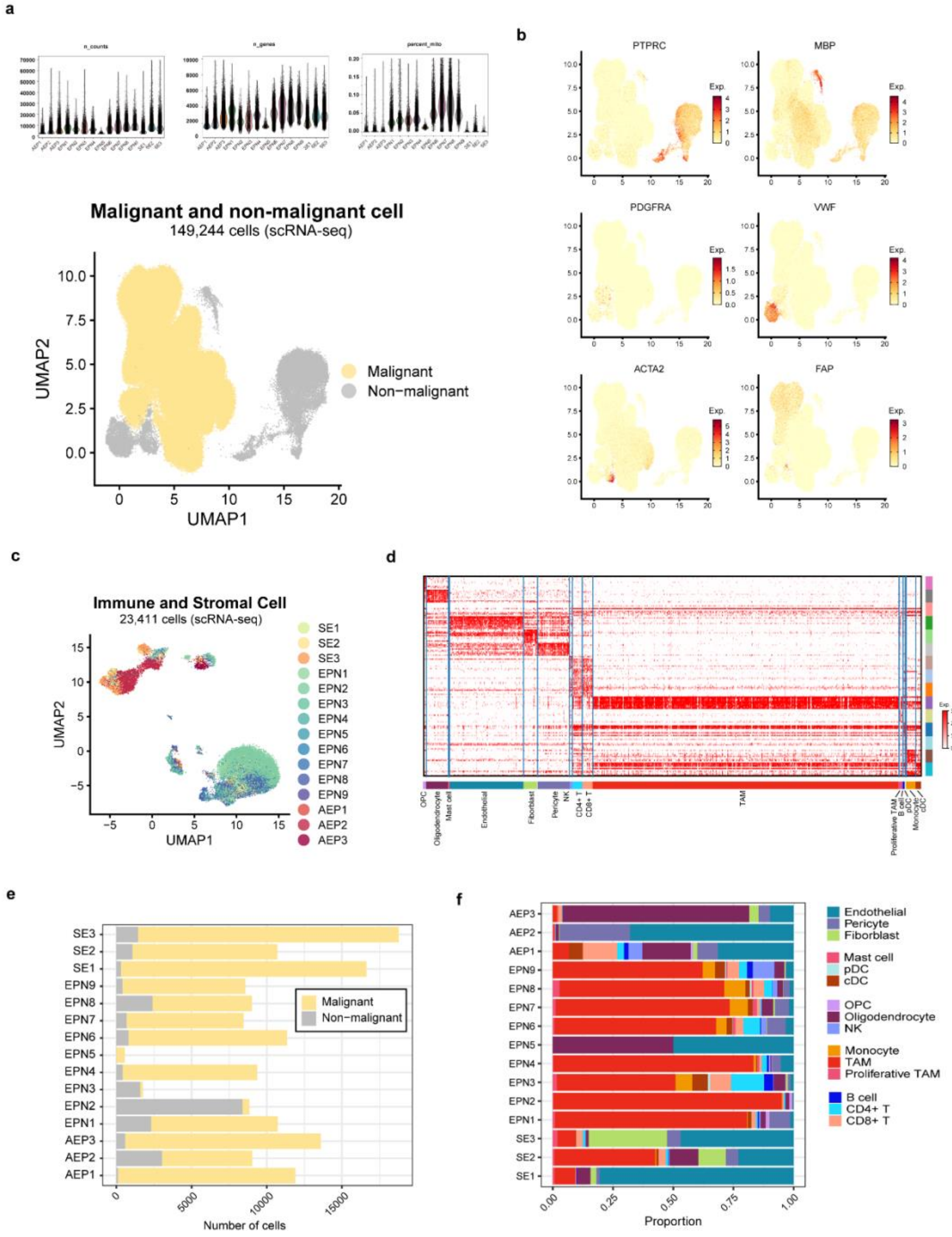

Supplementary Fig. 1 Cell Type Distribution in Spinal Ependymomas. Related to Fig. 1. (a) Quality control plot showing the remaining cells for analysis (top). UMAP plot showing both malignant and non-malignant cells in all samples, donor effect corrected by BBKNN (bottom). (b) UMAP plot showing the expression of stromal and immune cell marker genes. (c) UMAP plot showing the patient distribution of stromal

and immune cells, donor effect corrected by BBKNN. (d) Heatmap showing the expression of top10 DEGs of stromal and immune cell types. (e) Bar plot showing the number of malignant and non-malignant cells in each patient. Source data are provided as a Source Data file. (f) Bar plot showing the proportion of stromal and immune cells in each patient. Source data are provided as a Source Data file.

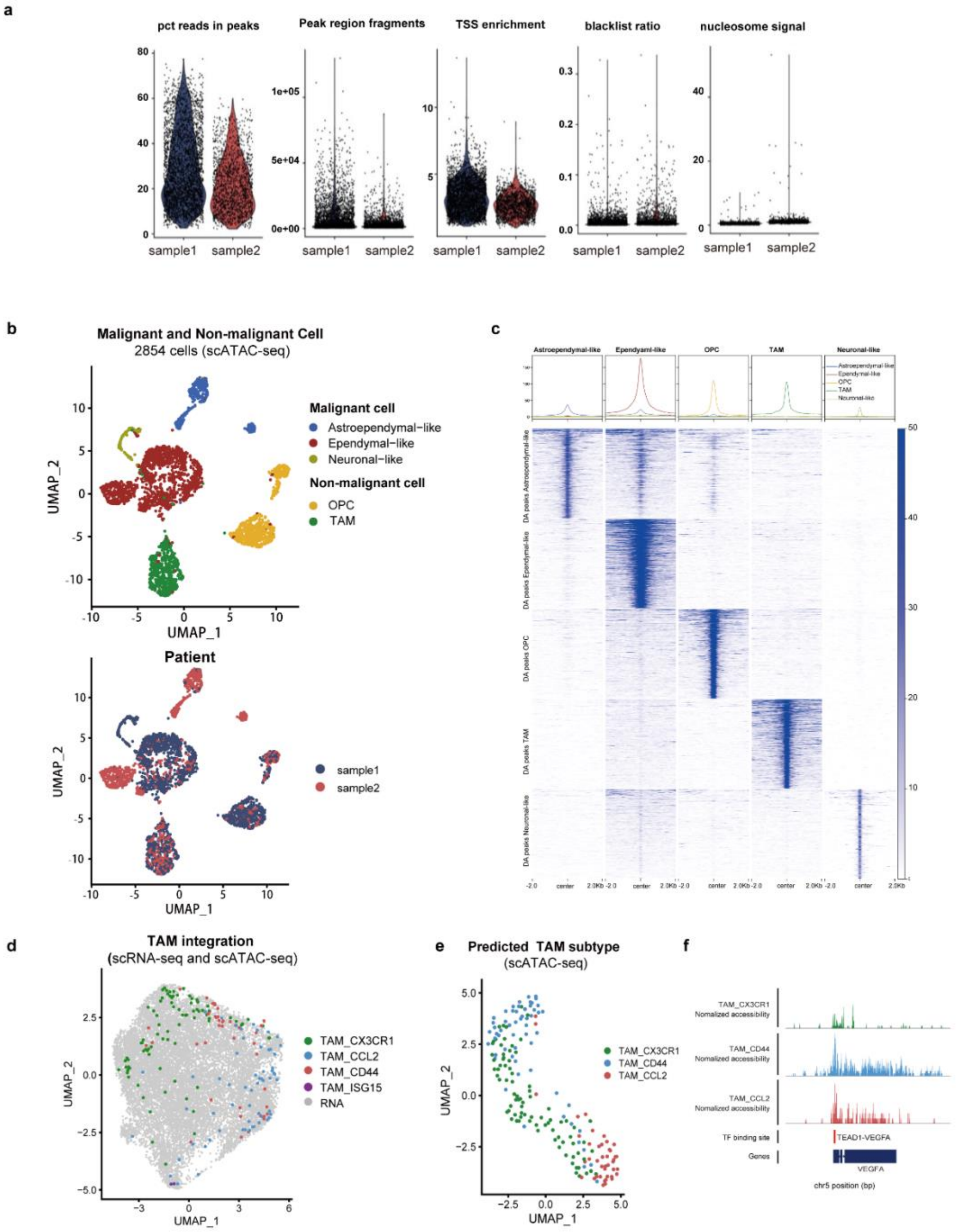

Supplementary Fig. 2 Subsets of Malignant cells and TAM in scATAC-seq. Related to Fig. 1 and Fig. 2. (a) Quality control plot for scATAC-seq experiment. Violin plot displaying the total number of fragments in peaks, fraction of fragments in peaks, TSS enrichment score, ratio reads in genomic blacklist regions and nucleosomal signal strength. (b) UMAP plot showing sub-clusters in scATAC-seq data (top) and patient distribution (bottom), donor effect corrected by Harmony. (c) Heatmap showing cluster-

specific ATAC-seq peaks for each cluster. (d) UMAP plot showing integration of scRNA-seq TAMs and scATAC-seq TAMs. (e) UMAP plot showing the predicted TAM sub-populations (3 major sub-clusters) in the scATAC-seq data. (f) Normalized ATAC-seq profile of *VEGFA* for TAM subpopulations and *TEAD1-VEGFA* binding site.

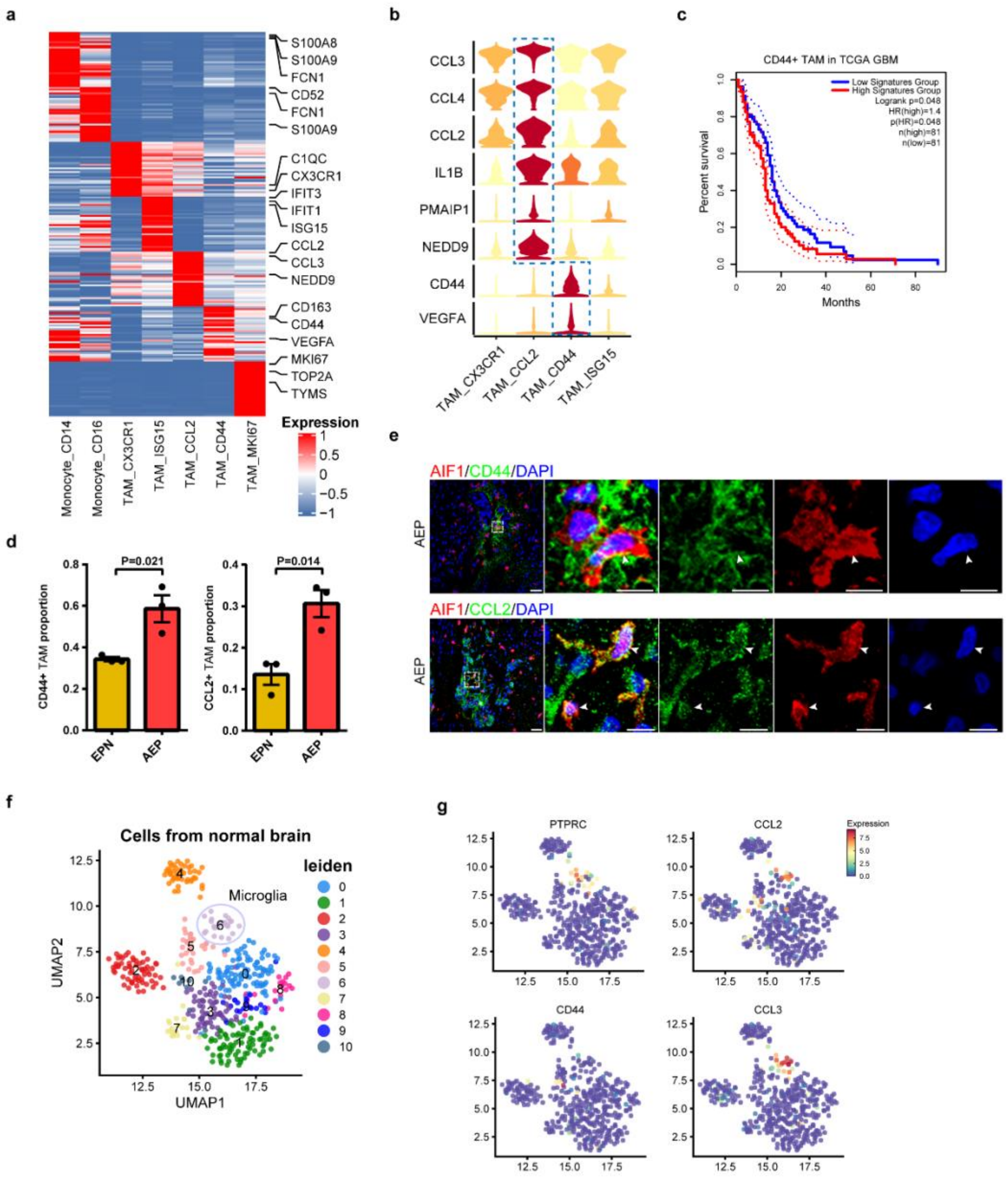

Supplementary Fig. 3 Subsets of Monocyte and TAM. Related to Fig. 2 and Fig. 3. (a) Heatmap showing signature genes for each monocyte and TAM subset. Selected genes are labeled on the right side. (b) Violin plot showing selected genes up-regulated in TAM subsets. (c) Kaplan-Meier plot showing worse clinical outcome for high expression of *CD44*$^{+}$ TAM signature genes in GBM patients from TCGA. +, censored observations. P-values were calculated by using both the log-rank test and Cox proportional hazards model. (d) Bar plots showing the proportion of *CCL2*$^{+}$ and *CD44*$^{+}$ TAMs in EPN and AEP (n =3 biologically independent samples). The P value was

calculated by T-test, Two-way ANOVA analysis. Data are presented as mean values +/- SEM. Source data are provided as a Source Data file. (e) Representative example of an AEP tumor stained by IF. The upper panel image indicates *AIF1*$^{+}$*CD44*$^{+}$ TAMs (the scale bar represents 30μm). The dashed boxes highlight regions shown on the right side and the arrow depicts the *CD44*$^{+}$ TAM in fluorescent images (the scale bar represents 100μm). The bottom panel image indicates *AIF1*$^{+}$*CCL2*$^{+}$ TAMs (the scale bar represents 30μm). The dashed boxes highlight regions shown on the right side and the arrow depicts the *CCL2*$^{+}$ TAMs in fluorescent images (the scale bar represents 100μm). Images shown are representatives of three independent experiments. (f) UMAP plot showing the cells from normal brain (reported by Darmanis, S. et al). Leiden cluster 6 marked in circle was Microglia. (g) UMAP feature plot showing the expression of microglia and TAMs marker genes in normal brain cells.

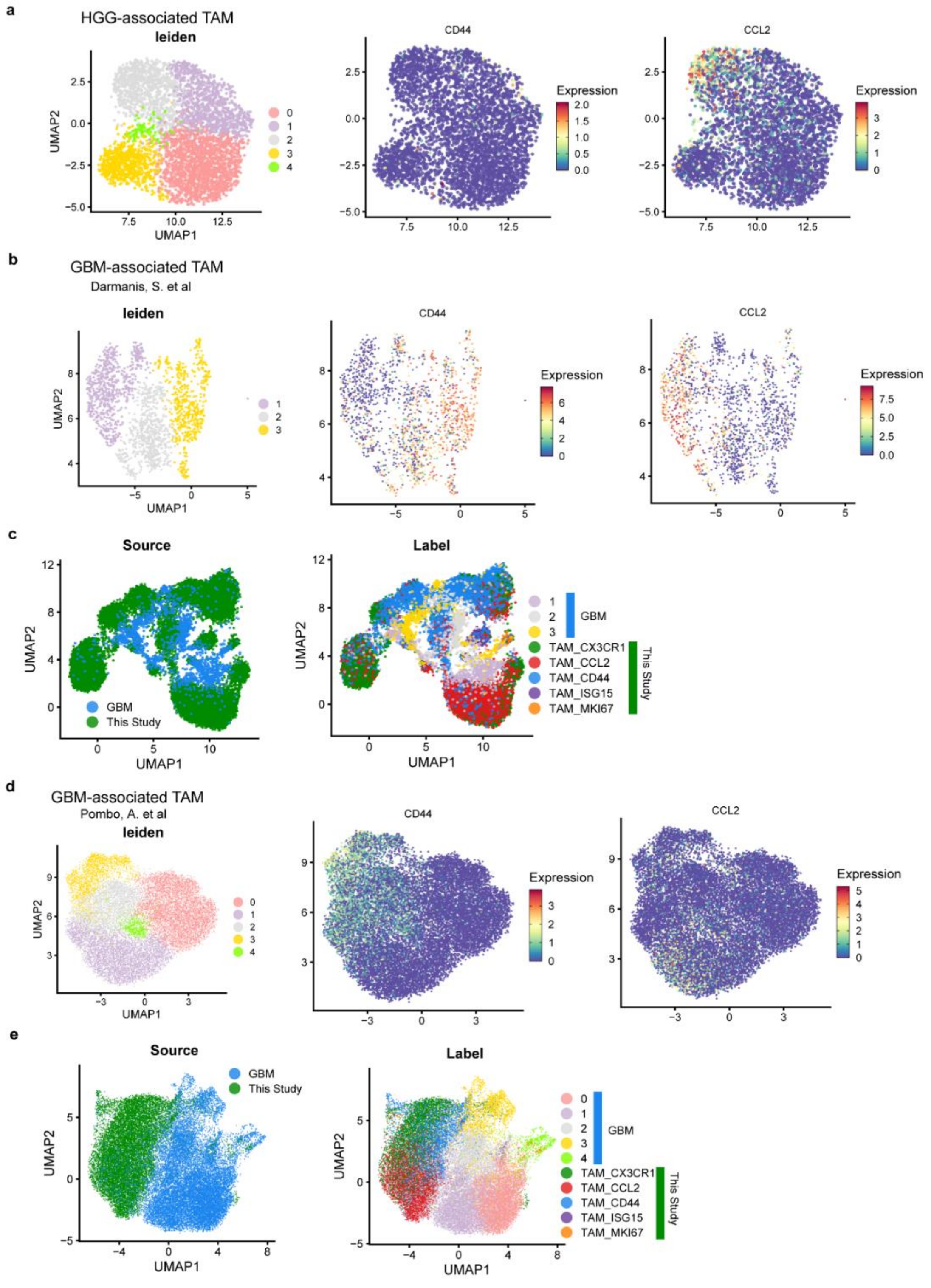

Supplementary Fig. 4 Comparison of TAMs from Spinal Ependymoma and Other Neurological Tumors. Related to Fig. 2 and Fig. 3. (a) UMAP plot showing the major TAM subsets from HGG (reported by Friedrich et al) (left). Feature plot showing the *CD44* and *CCL2* expression in TAM subsets (right). (b) UMAP plot showing the major

TAM subsets from GBM (reported by Darmanis, S. et al) (left). Feature plot showing the *CD44* and *CCL2* expression in TAM subsets (right). (c) UMAP plot showing the integration of TAMs subsets and source origin from GBM (reported by Darmanis, S. et al) and spinal ependymoma. (d) UMAP plot showing the major TAM subsets from GBM (reported by Pombo, A. et al) (left). Feature plot showing the *CD44* and *CCL2* expression in TAM subsets (right). (e) UMAP plot showing the integration of TAMs subsets and source origin from GBM (reported by Pombo, A. et al) and spinal ependymoma.

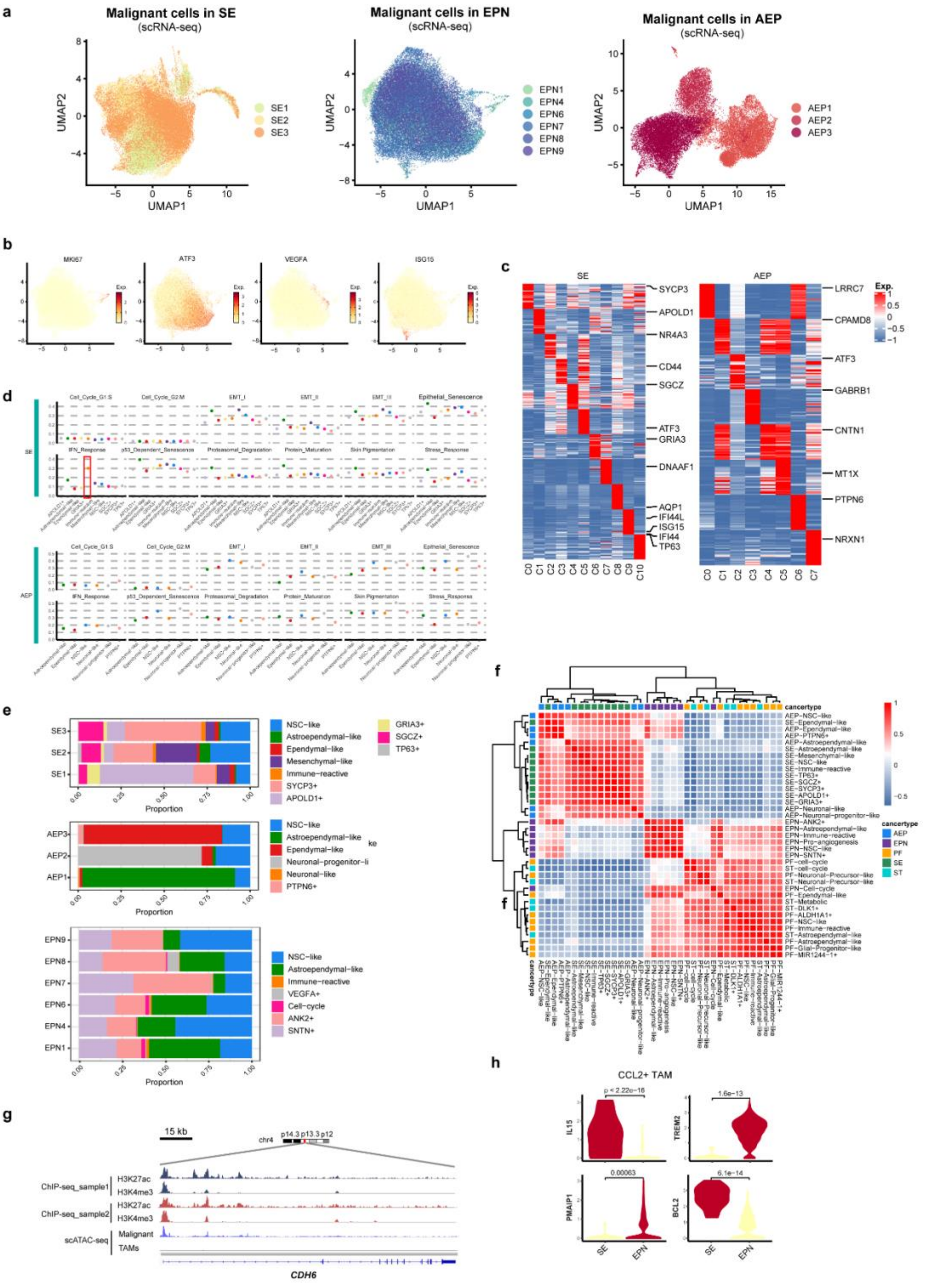

Supplementary Fig. 5 Heterogeneity of Malignant Cells in Each Ependymoma Cancer Subtype. Related to Fig. 4 and Fig. 5. (a) UMAP plot showing the patient distribution of malignant cells in three type cancer, donor effect corrected by Harmony. (b) UMAP plots showing expression levels of selected genes in EPN. (c) Heatmaps

showing signature genes for each malignant cell sub-cluster in SE and AEP. Selected genes were labeled on the right side. (d) Scatter plots showing average expression of 12 generic tumor cell programs across each malignant subsets in SE and AEP. (e) Bar plots showing the proportion of each malignant subset in each patient. Source data are provided as a Source Data file. (f) Heatmap showing comparison of malignant cells from spinal, supratentorial and posterior fossa ependymoma. SE, EPN, AEP are spinal ependymomas in our study, PF is posterior fossa ependymoma, ST is supratentorial ependymomas. (g) Browser tracks showing epigenetic signals around *CDH6* in ChIP-seq and scATAC-seq. (h) Violin plots showing selected DEGs in *CCL2*$^{+}$ TAMs across different cancer subtypes. We excluded *CCL2*$^{+}$ TAMs in AEP because of the limited cell number (N=1). P-values were calculated by Wilcoxon test, two-sided comparisons.

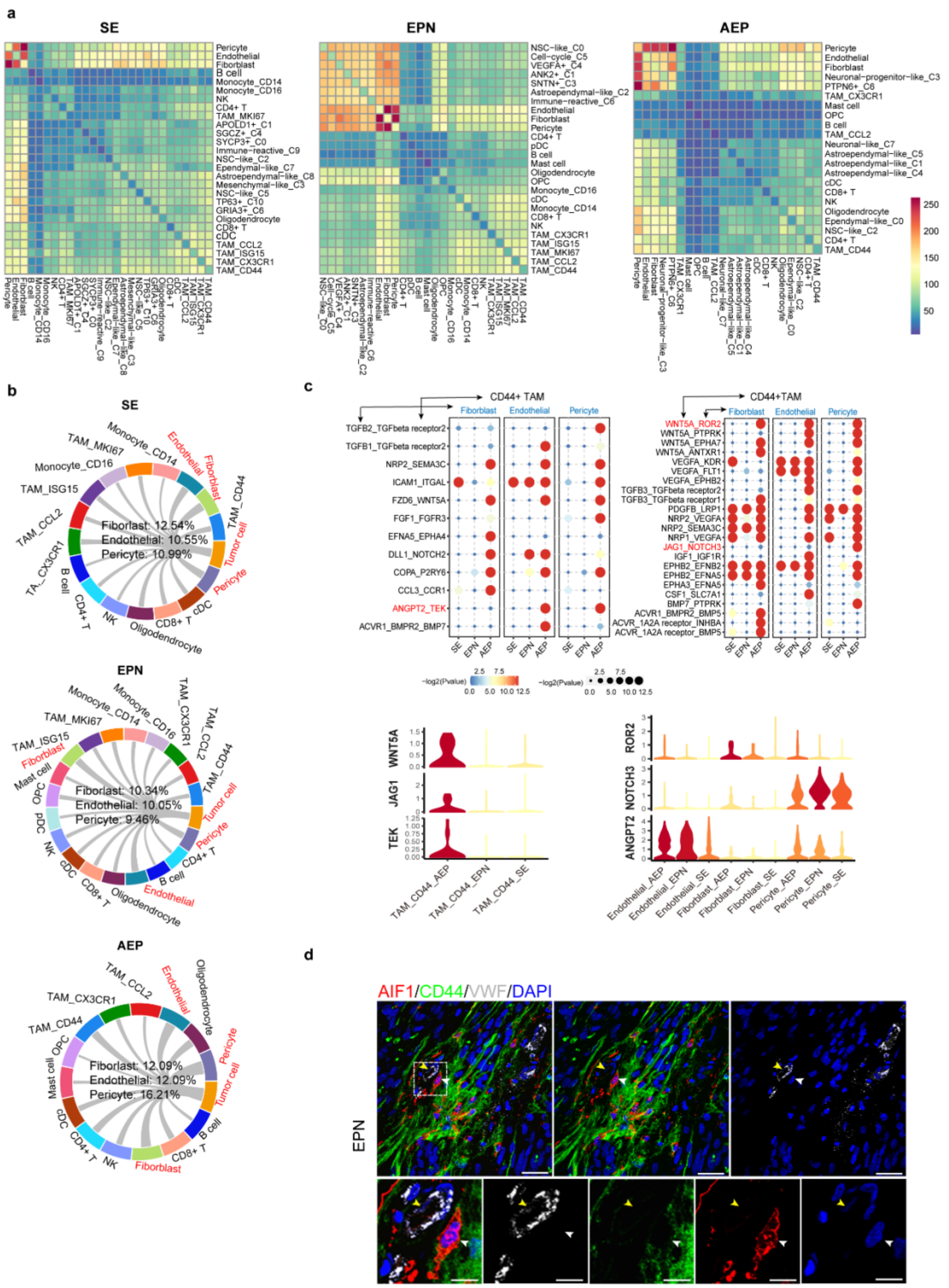

Supplementary Fig. 6 Cell-cell Interaction Analyses for *CD44*⁺ TAMs. Related to Fig. 6. (a) Heatmaps showing the total number of interaction events between cell types in each cancer subtype reported by CellPhoneDB. (b) Circos plot showing putative ligand-receptor interactions between tumor cells and non-malignant cells. The fractions of significant interaction events between tumor cells and fibroblasts/endothelial

cells/pericytes are summarized. The fraction of each interaction pair was calculated by dividing the total number of interaction events related to tumor cells in each cancer subtype. (c) Bubble heatmap showing selected LR pairs between *CD44*$^{+}$ TAMs and stromal cells (including fibroblasts, endothelial cells and pericytes) in each cancer subtype. Each row represents an LR pair, and each column defines a cell-cell interaction pair in a specific cancer subtype. P-values were indicated by circle color and size (top). P-values were calculated by CellPhoneDB. Violin plot showing the expression level of the chosen ligand-receptor pairs which was marked red in the bubble heatmap (bottom). (d) Representative example of an EPN tumor stained by IF with anti-AIF1 (red), CD44 (green), VWF (gray) and DAPI (blue) antibodies. The dashed box highlights the region shown in the bottom panel. The white arrow depicts the *CD44*$^{+}$ TAMs and the yellow arrow depicts the endothelial cells in fluorescent images. The scale bar in the top panel represents 30μm and the scale bar in the bottom panel represents 100μm. Images shown are representatives of three independent experiments.

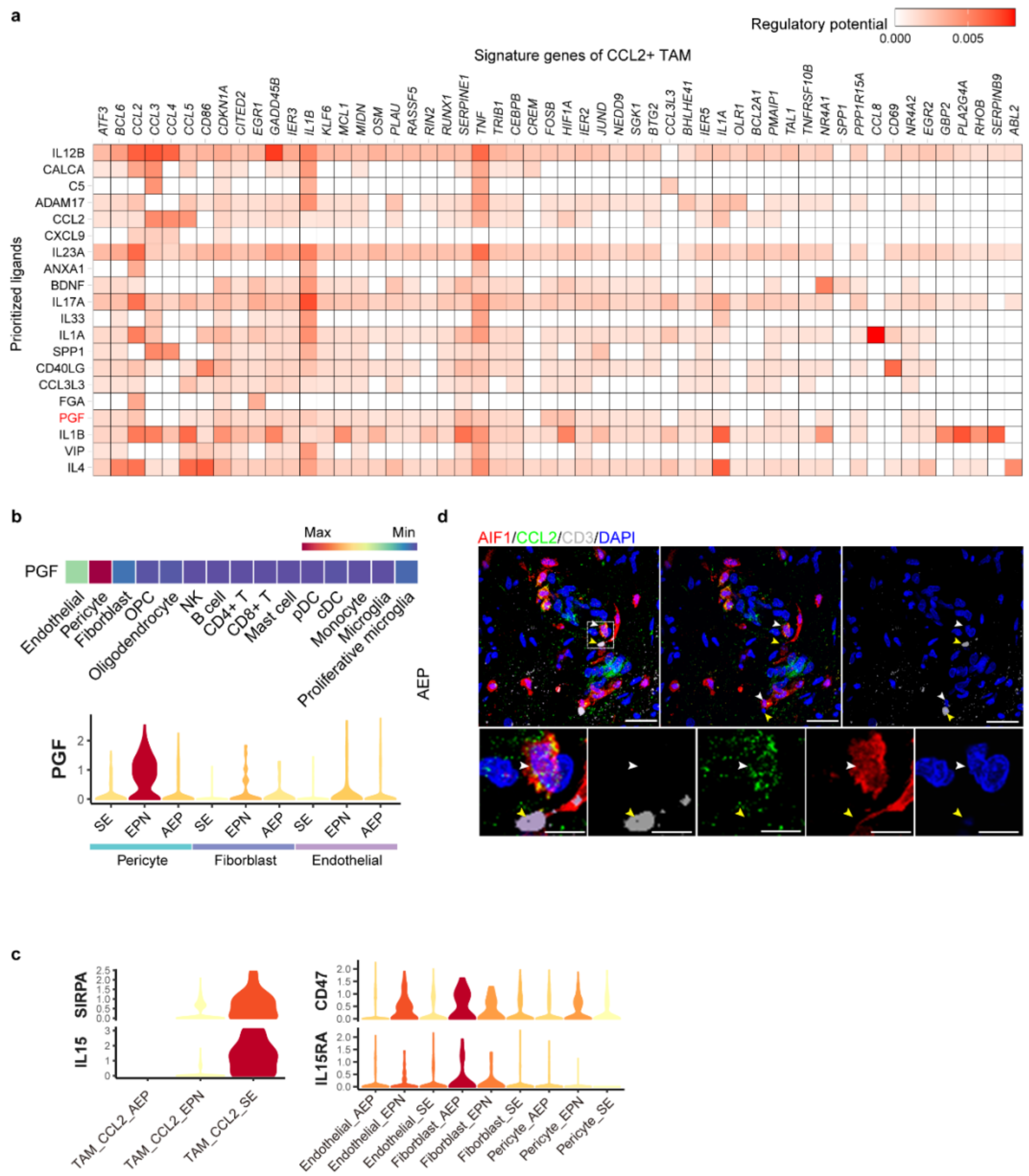

Supplementary Fig. 7 Cell-cell Communication Analyses for *CCL2*⁺ TAMs. Related to Fig. 6. (a) Heatmap showing potential ligands driving the signature of *CCL2*⁺ TAMs. (b) Heatmap showing the expression of *PGF* in stromal and immune cells (top). Violin plot showing the expression of *PGF* in fibroblasts, endothelial cells and pericytes from each cancer subtype (bottom). (c) Violin plot showing the expression level of the chosen ligand-receptor pairs which was marked red in **Fig. 6f**. (d) Representative example of an AEP tumor stained by IF with anti-AIF1 (red), CCL2 (green), CD3 (gray) and DAPI (blue) antibodies. The white arrow depicts the *CCL2*⁺ TAMs and the yellow arrow depicts the T cells in fluorescent images. The scale bar in

the top panel represents 30µm and the scale bar in the bottom panel represents 100µm. Images shown are representatives of three independent experiments.
