# Supplementary material for: Interrogation of the microenvironmental landscape in spinal ependymomas reveals dual functions of tumor-associated macrophages
Source: Nat Commun. 2021 Nov 25;12:6867. doi: 10.1038/s41467-021-27018-9 (PMC8617028; doi:10.1038/s41467-021-27018-9)
Supplement: Supplementary file 2 — Description of Additional Supplementary Files [file 41467_2021_27018_MOESM2_ESM.pdf]

Description of Additional Supplementary Files

Title: Supplementary Data 1.

Description: Summary of clinical information for patients collected in this study. Related to **Fig. 1**.

Title: Supplementary Data 2.

Description: Signature genes of each non-malignant cells subset. Related to **Fig. 1**.

Title: Supplementary Data 3.

Description: Signature genes of TAM subsets. Related to **Fig. 2**.

Title: Supplementary Data 4.

Description: Hallmark pathway enrichment analyses items. Related to **Fig. 2, Fig. 5.** Adjusted p-values were calculated by using *enrichr* function from R package clusterProfile with hypergeometric test statistical analyses.

Title: Supplementary Data 5.

Description: Summary of differentially accesible peaks between TAM subtypes. Related to **Fig. 2.** For computing differentially accessible peaks among distinct clusters, statistical analyses Wilcoxon Rank Sum test were performed using function *FindAllMarkers* of Seurat.

Title: Supplementary Data 6.

Description: Signature genes of each malignant subset in three type cancer. Related to **Fig. 4.**

Title: Supplementary Data 7.

Description: Targetable genes in malignant cells in each cancer subtypes. Related to **Fig. 4**.

Title: Supplementary Data 8.

Description: Differently expression genes in malignant cells from different cancer subtypes. Related to **Fig. 5**. Statistical analyses Wilcoxon test were performed using function *rank_genes_groups* of SCANPY.

Title: Supplementary Data 9.

Description: Intersection of EMT-related genes with active transcriptional signals. Related to **Fig. 5.**

Title: Supplementary Data 10.

Description: Differently expression genes in TAMs from different cancer subtypes. Related to **Fig. 5**. Statistical analyses Wilcoxon test were performed using function *rank_genes_groups* of SCANPY.
